# Multimodal learning in clinical proteomics: enhancing antimicrobial resistance prediction models with chemical information

**DOI:** 10.1093/bioinformatics/btad717

**Published:** 2023-11-24

**Authors:** Giovanni Visonà, Diane Duroux, Lucas Miranda, Emese Sükei, Yiran Li, Karsten Borgwardt, Carlos Oliver

**Affiliations:** Department of Empirical Inference, Max Planck Institute for Intelligent Systems, Max-Planck-Ring 4, Tübingen 72076, Germany; BIO3—GIGA-R Medical Genomics, University of Liège, Avenue de l’Hôpital 11, Liège 4000, Belgium; ETH AI Center, ETH Zürich, Andreasstrasse 5, Zürich 8092, Switzerland; Research Group Statistical Genetics, Max Planck Institute of Psychiatry, Kraepelinstraße 10, München 80804, Germany; Department of Signal Theory and Communications, Universidad Carlos III de Madrid, Leganés 28911, Spain; Department of Biosystems Science and Engineering, ETH Zürich, Basel 4058, Switzerland; Department of Biosystems Science and Engineering, ETH Zürich, Basel 4058, Switzerland; Swiss Institute for Bioinformatics (SIB), Amphipôle, Quartier UNIL-Sorge, Lausanne 1015, Switzerland; Department of Machine Learning and Systems Biology, Max Planck Institute of Biochemistry, Martinsried 82152, Germany; Department of Biosystems Science and Engineering, ETH Zürich, Basel 4058, Switzerland; Swiss Institute for Bioinformatics (SIB), Amphipôle, Quartier UNIL-Sorge, Lausanne 1015, Switzerland; Department of Machine Learning and Systems Biology, Max Planck Institute of Biochemistry, Martinsried 82152, Germany

## Abstract

**Motivation:**

Large-scale clinical proteomics datasets of infectious pathogens, combined with antimicrobial resistance outcomes, have recently opened the door for machine learning models which aim to improve clinical treatment by predicting resistance early. However, existing prediction frameworks typically train a separate model for each antimicrobial and species in order to predict a pathogen’s resistance outcome, resulting in missed opportunities for chemical knowledge transfer and generalizability.

**Results:**

We demonstrate the effectiveness of multimodal learning over proteomic and chemical features by exploring two clinically relevant tasks for our proposed deep learning models: drug recommendation and generalized resistance prediction. By adopting this multi-view representation of the pathogenic samples and leveraging the scale of the available datasets, our models outperformed the previous single-drug and single-species predictive models by statistically significant margins. We extensively validated the multi-drug setting, highlighting the challenges in generalizing beyond the training data distribution, and quantitatively demonstrate how suitable representations of antimicrobial drugs constitute a crucial tool in the development of clinically relevant predictive models.

**Availability and implementation:**

The code used to produce the results presented in this article is available at https://github.com/BorgwardtLab/MultimodalAMR.

## 1 Introduction

Antimicrobial resistance (AMR) poses a significant threat to human health worldwide. Based on recently published predictive statistical models, an estimated 4.95 million (3.62–6.57) deaths were associated with bacterial AMR in 2019, including 1.27 million (95% UI 0.911–1.71) deaths attributable to bacterial AMR (Murray *et al.* 2022). Effective prevention strategies are urgently needed to stall AMR emergence and dissemination.

With a detailed understanding of the potential resistance mechanisms of the pathogen, clinicians can select specific antimicrobials with a higher chance of success. In this regard, disk-diffusion and microdilution antibiograms are still the references for determining AMR (Benkova *et al.* 2020). While effective, these approaches are too cumbersome and time-consuming to enable the rapid selection of an adequate targeted antimicrobial treatment (Barlam *et al.* 2016, Arena *et al.* 2017, Feucherolles *et al.* 2022).

The emergence of matrix-assisted laser desorption/ionization time-of-flight mass spectrometry (MALDI-TOF MS) provides a fast and cost-effective method for analysing bacterial strains. This technology is predominantly used as an analytical tool to identify and understand the structure of unknown biomolecules (Bookstaver *et al.* 2017, Mangioni *et al.* 2019, Han *et al.* 2021), and it has been used as an AMR detection tool in the clinic (De Carolis *et al.* 2014). However, the usefulness of MALDI-TOF as a data source for machine learning (ML) AMR detection has only recently garnered interest in research (Weis *et al.* 2020, Goodswen *et al.* 2021, Kim *et al.* 2022). These studies have mainly focussed on creating models for specific combinations of antimicrobials and pathogens.

Many state-of-the-art (SOTA) tools such as CARD-RGI (Jia *et al.* 2016), AMRFinder (Feldgarden *et al.* 2019), and SARGFAM (Yin *et al.* 2018) use variants of alignment-based methods like BLAST (Altschul *et al.* 1990). More recently, deep learning-based techniques have shown SOTA performance. Using similarity features to compare the query sequence to existing antimicrobial resistance gene (ARG) databases, DeepARG (Arango-Argoty *et al.* 2018) was developed by building on a multi-layer perceptron model. Li *et al.* (2021) proposed a multitask deep learning framework called HMD-ARG that first predicts whether the input sequence is an ARG and then predicts the resistant antimicrobial family, resistance mechanism, and gene mobility. Many published studies used pathogens such as *Staphylococcus aureus* and the β-lactam antimicrobial family (Sogawa *et al.* 2017, Wang *et al.* 2018, Tang *et al.* 2019). Other relevant clinical pathogens, such as quinolones and macrolides, were studied in Sabença *et al.* (2020) and Sousa *et al.* (2020). Feucherolles *et al.* (2022) show that MALDI-TOF MS combined with ML provides a useful tool for AMR screening in the case of *Campylobacter coli* and *C.jejuni*.

In 2022, Weis *et al.* (2022) developed a large (over 700 000 resistance labels and 300 000 MALDI-TOF spectra) ‘Database of Resistance Information on Antimicrobials and MALDI-TOF Mass Spectra (DRIAMS)’ and utilized ML models to predict the resistance of significant pathogens like *S.aureus*, *Escherichia coli*, and *Klebsiella pneumoniae*. The study concluded that focussing on predicting resistance for specific species–drug pairs improved classifier accuracy, likely due to the complexity of resistance mechanisms.

Drug recommendation is another ML application that has been gaining significant interest, particularly in cancer research. Various solutions have emerged, including the Kernelized Bayesian Multi-Task Learning (Gönen and Margolin 2014), which learns the relationships between different drugs during training. This algorithm, along with random forest, was the best-performing approach in a challenge-based competition on a breast cancer dataset (Costello *et al.* 2014). Another promising approach is Kernelized Rank Learning (He *et al.* 2018), which focusses on providing a ranked list of drugs instead of exact sensitivity values and was specifically designed to handle sparse training datasets. Along these lines, recommendation models could assist in maintaining or enhancing infection coverage rates while employing fewer broad-spectrum antimicrobials than current practices (Corbin *et al.* 2022).

Although previous works show promising results, the current SOTA AMR prediction methods based on proteomics do not integrate multiple relevant data sources, such as the chemical composition of antimicrobials alongside MALDI-TOF spectra obtained from pathogenic samples. Instead, a separate model is trained for each antimicrobial and pathogen species combination, limiting the potential for knowledge transfer, generalizability to new drugs, and deciphering the underlying resistance mechanisms. Developing such general-purpose models could enhance patient care in a robust and highly adaptable way.

**Figure 1.**
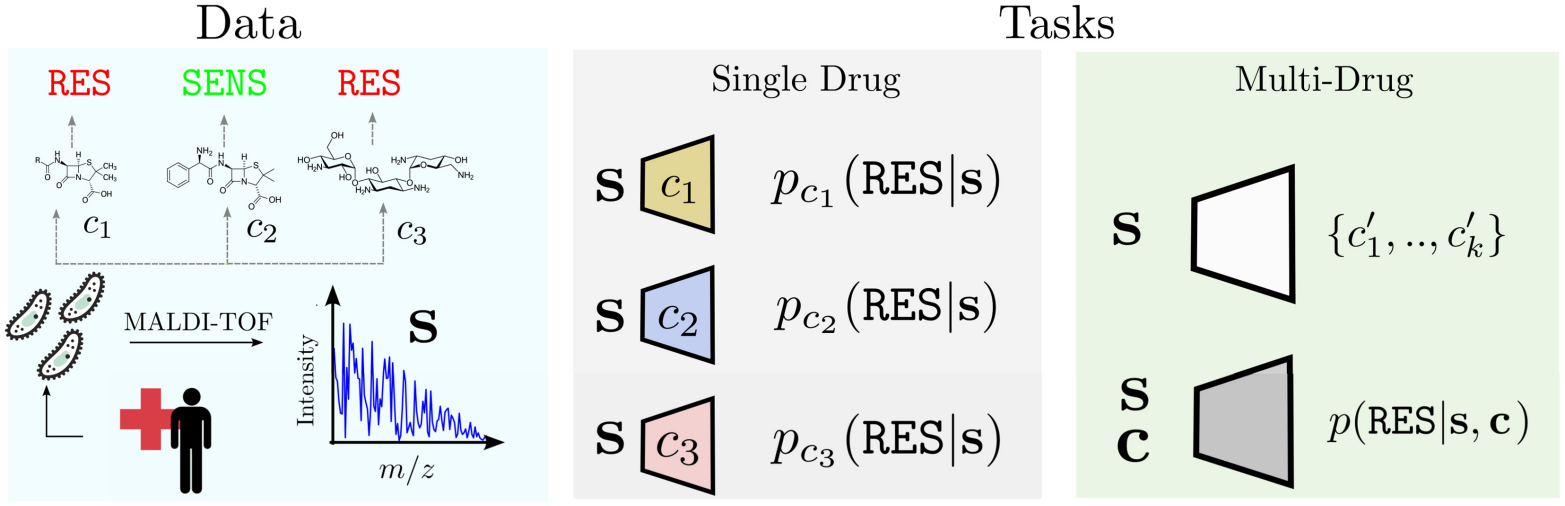
Description of antimicrobial resistance prediction tasks. The dataset consists of MALDI-TOF mass spectra for bacterial samples of hospital patients treated for infection. For each sample, a set of compounds is annotated as inducing a sensitive or resistant outcome in the bacterial sample (left panel). From this data, we construct two new tasks extending the previous setting (middle panel) where each compound gives rise to a single binary classification task of resistance versus sensitivity for a given spectrum s. In this work, we introduce two tasks (right panel), which are to ‘predict resistance’ given a drug–spectrum pair and to ‘recommend’ antimicrobials for a given observed spectrum.

To address this problem, our work focusses on incorporating chemical data into AMR prediction using mass spectrometry pathogen profiles (Fig. 1). This learning framework has been successfully applied in predicting cell line response to cancer drugs, with some models proving successful (Chiu *et al.* 2019, Baptista *et al.* 2021). We propose several prediction and evaluation settings for AMR where chemical information can be utilized and demonstrate increased prediction performance and generalizability compared to single-drug models. Furthermore, we define direct drug recommendation models to predict drugs with a high chance of sensitivity or resistance for unseen spectra and thoroughly evaluate their feasibility and performance.

## 2 Materials and methods

Using the DRIAMS dataset (Section 2.1.1) and molecular fingerprinting (Section 2.1.2), we explore two major prediction settings which leverage chemical information: drug recommendation (Section 2.2) and resistance prediction (Section 2.3). Through these settings, we test whether chemical information can be used to improve resistance prediction SOTA and propose new model development avenues. These workflows are illustrated in Fig. 1.

### 2.1 Dataset

#### 2.1.1 MALDI-TOF mass spectra dataset

This study utilized the publicly accessible DRIAMS dataset (Weis *et al.* 2021), a comprehensive resource comprising MALDI-TOF mass spectra obtained from hospital patients across four Swiss diagnostic labs during the period spanning 2016–2018. The dataset encompasses 303 195 mass spectra and 768 300 AMR labels, covering 803 different bacterial and fungal pathogen species. The dataset has been meticulously organized into four distinct subcollections, each representing different hospital sites. Each data point contains a mass spectrum from a patient sample, complemented by annotations denoting its susceptibility or resistance to as many as 71 antimicrobials. In our analysis, we harnessed the 6000D binned mass spectra vector representation, aligning with the methodology proposed by Weis *et al.* (2022).

#### 2.1.2 Chemical feature extraction

Molecular fingerprinting (Willett *et al.* 1998, Bajorath 2001) is a popular method for encoding chemical information into numerical features for ML models. It represents a molecule as a series of bits that encode the presence or absence of certain substructures. This technique captures important information about the molecular structure, including topological, physio-chemical, and structural properties. The use of molecular fingerprints is prevalent in chemical informatics and drug discovery and has been shown to be effective in many applications (David *et al.* 2020).

We tested three standard techniques, namely the Molecular ACCess Systems keys fingerprints (MACCS) (Durant *et al.* 2002) (166-bit keyset), the PubChem Fingerprints (PubChemFP) (Wang *et al.* 2017) (881-bit long keys), and the 1024 bits long Morgan fingerprints (Morgan 1965). We obtained the fingerprints for the antimicrobial drugs in the DRIAMS dataset using RDKit (Landrum *et al.* 2022), and PubChemPy (Swain 2014), two open-source Python packages. Certain treatments present in the dataset consist of mixtures of compounds; since it is impossible to associate a fingerprint representation in such cases, they have been excluded from our analysis.

### 2.2 Drug recommendation

We first examine the interaction between clinical proteomics and chemical features through the task of drug recommendation. In the ‘recommendation’ setting, a model directly suggests a set or a ranking of potentially suitable drugs for a query spectrum. To perform this search, we test various explicit and learned functions of spectrum and chemical similarity for each query spectrum, returning *n* recommendations.

We evaluate the effectiveness of five personalized treatment recommendation methods focussing on the impact of incorporating various levels of information into the drug ranking process, including the pathogen species, spectra, and drug features:

1. Random baseline: randomly select *k* samples from the training set for a query sample and return the drugs that most frequently elicited a sensitive reading among the *k* samples.
2. Baseline species: randomly select *k* samples from the training set which correspond to the same pathogen species as the query and again return the drug which most frequently results as effective.
3. Spectrum similarity: given a similarity function between spectra, select the *k* most similar spectra to the query. We test multiple measures of similarity between spectra, namely cosine similarity, correlation, Euclidean, Manhattan, and Wasserstein distances.
4. Siamese networks: learn joint embeddings of the drugs and spectra and use logistic regression (LR) on the embeddings to rank drugs for recommendations based on the resulting probabilities.
  Siamese networks (Chicco 2021) contain two identical subnetworks with shared weights and work in tandem on two input vectors composed of the MALDI-TOF mass spectra and the chemical fingerprints to minimize the difference between the actual and predicted similarity between pairs of observations (Supplementary Fig. S1a).
5. ResMLP: train a classification Multi-Layer Perceptron with Residual Skip-Connections (Szegedy *et al.* 2017) network to predict the probability of resistance for drug–spectrum pairs. Each drug is then ranked according to the predicted resistance likelihood. This model uses skip-connections that provide a path for data to reach deeper layers in the network by skipping some layers (Supplementary Fig. S1b), generally improving the training procedure. To account for the different number of features in the MALDI-TOF mass spectrum and the chemical fingerprint, the model first projects each to the same dimension before concatenating the two vector representations and using them as input for the feed-forward network.

We test multiple values of k∈{1,…100} and study their impact on performance to determine the optimal threshold. Then, we use majority voting: among the drugs with known response values for the test sample, we recommend the drug that results most often sensitive across the chosen *k* samples. If multiple drugs have the highest occurrence, we select all as recommended treatments. If there are no common drugs between the drugs tested for a specific sample in the test and the drugs we recommend, then we do not compute the performance.

#### 2.2.1 Evaluation

The test set consists of a random selection of 20% of the samples and all associated tested drugs to ensure that all observations related to a spectrum are in the same set. Additionally, we impose a constraint that each spectrum in the testing set must be associated with at least one resistant and one sensitive outcome.

We conduct our recommendation analyses on the DRIAMS-B dataset, with the training set containing 1907 samples and the test set containing 477 samples, and report multiple measures to evaluate the performance of each approach derived from the literature on information retrieval, namely Precision *P*, Precision at cutoff *n* P@n, and the mean Average Precision at cutoff *n* mAP@n (additional details in the Supplementary Material).

### 2.3 Generalized AMR prediction

In the resistance prediction task, each observation corresponds to a biological sample and a drug to which it was exposed. The aim is to associate with each sample–drug pair an outcome that estimates the likelihood of the sample being resistant to the drug.

The task can be formalized as learning a mapping f:X×C→[0,1], where X is the space of bacterial samples and C is the space of chemical compounds. Each instance is represented by the measured MALDI-TOF spectrum, while the chosen molecular fingerprints represent the antimicrobial drugs. With this formalism, we model the output $f(x_{i},c_{j})$ as the probability that the sample corresponding to the mass spectrum $x_{i}$ is resistant to the antimicrobial drug represented by $c_{j}$. This formalism generalizes the original prediction setting introduced in Weis *et al.* (2022), which learns one predictor for each compound and only uses the spectrum as input.

We compared three ML-based approaches to model the resistance prediction function:

1. Baseline: principal component analysis (PCA) with LR. As the dimensions of the mass spectra and the chemical fingerprints are of different scales, applying PCA projects them to lower and comparable dimensions while preserving 95% of the variance of the original variables. These embeddings were then concatenated and used as input for the LR model.
2. Siamese networks: similarly to the drug recommendation case, we use the learnt joint representations from the Siamese networks as input to LR to yield the resistance predictions.
3. ResMLP: train a classification ResMLP to predict the probability of resistance (we use the same configuration as the recommendation task; see Section 2.2).

We designed three data splits to reflect different data-generating processes to examine the prediction capabilities of the previously described ML models.

1. Random split: the observations in each DRIAMS dataset are randomly sampled to create training, validation, and test sets with a partitioning of 70%, 10%, and 20%, respectively. This data split corresponds to the i.i.d. setting, where all the sets are drawn from the same joint distribution.
2. Species-drug zero-shot split: the test set contains novel ‘pairs’ of species and drugs. Given the finite size of the datasets, we used a heuristic to randomly select species–drug combinations that account for approximately 20% of the data and ensured that the species *s* and the drug *d* are not present in any observations of the training set. The remaining data are randomly split into training and validation sets that contain approximately 70% and 10% of the dataset, respectively.
3. Drug zero-shot split: we hold out as a test set all the observations corresponding to the target drug *d* and test how accurate the predicted resistances are for a compound that the model has never seen in training.

We report three standard classification metrics for imbalanced data: area under the precision-recall curve (AUPRC), balanced accuracy, and Matthews Correlation Coefficient (MCC). To analyse the importance of chemical features in the AMR prediction task, we employed SHAP (Lundberg and Lee 2017), a framework rooted in game theory that is among the most popular *post hoc* interpretation methodologies (additional details in the Supplementary Material).

## 3 Results

In this section, we ask whether (i) the drug recommendation setting using DRIAMS contains useful and nontrivial spectrum–drug associations (Sections 3.1 and 3.2) and (ii) whether generalized AMR prediction models are able to use chemical information to significantly outperform SOTA single-drug models (Sections 3.3, 3.4, and 3.5).

### 3.1 Model-free approaches offer strong baselines for recommending AMR drugs

We first analyse the use of three model-free recommendation approaches to select drugs suitable for treating clinical patients (Section 2.2). The ‘random baseline’, ‘baseline species’, and ‘spectrum similarity’ methodologies rely on similarities between a test sample and samples from the training set. In these set-ups, we select the top-*k* similar samples and recommend the drugs that result as effective most often in the selected set.

**Figure 2.**
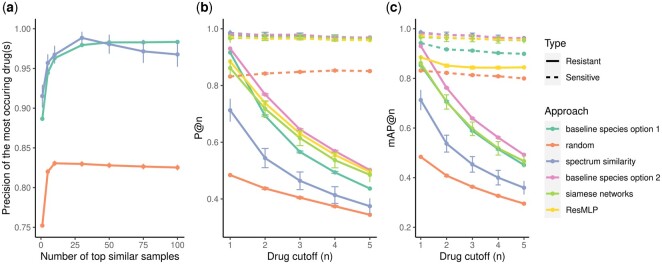
(a) Recommendation performance from the top-*k* similar samples, with 95% confidence intervals: the top-*k* most similar samples to the new observation are selected. Drugs are ranked according to the number of similar samples sensitive to the drugs. The drug that exhibits the highest frequency of sensitivity is identified. If multiple drugs show comparable sensitivity properties across similar samples, we include them all. The precision obtained from these drugs for the new sample is reported. Three approaches to assess the similarity between samples are compared: ‘random’, ‘Baseline Species Option 1’, and ‘Spectrum Similarity’ (details in Section 2.2). Precision (b) and mean average precision (c) at multiple cut-offs *n* across all samples in the test set for the different recommendation methods. For the ‘random baseline’, ‘baseline species’, and ‘spectrum similarity option 1’ approaches, the number of top neighbours *k* is set to 30. In the ‘spectrum similarity option 2’ set-up, *k* is the maximum number of samples available in the training set that correspond to the same species that the sample investigated in the testing set. The dashed lines in (b) and (c) represent recommendations that aim to assign high ranks to drugs to which the sample is sensitive, while the continuous lines aim to rank the drugs to which the sample is resistant.

For all three methods, the test precision quickly increases up to 15≤k≤30, then stabilizing or showing small changes (Fig. 2a). Therefore, selecting a high *k* is preferred over including only a few samples. Based on these results, in the following analyses, we used k=30.

The performance based on the ‘random baseline’ set-up is the lowest among the three methods. This indicates that the other two methods incorporate additional information beyond recommending drugs based on the highest occurrence across samples. The ‘spectrum similarity’ approach led to comparable performance to the ‘baseline species’ method, suggesting that spectra similarity is insufficient to capture significant additional information compared to the species. In the ‘baseline species’, the performance is only computed when more than *k* available samples correspond to the same species in the training set. This could introduce a bias when *k* increases if the number of species in the training set is not random but can be accounted for by external variables or properties. For instance, the performance could be deflated if the drug sensitivity is more homogeneous for species that only appear a few times in the dataset.

We evaluated the effect of increasing the number of top similar samples on the number of recommended drugs. Indeed, if multiple drugs have the same highest occurrence across the top-similar samples, it leads to the recommendation of several drugs. We found that as the number of samples used for the majority vote increases, the likelihood of obtaining a ranking with no similar occurrence also increases (Supplementary Fig. S11a). The ‘baseline species’ set-up resulted in the highest number of recommendations (ranging from 12 drugs with k=1 to 2 drugs with k=100) while the ‘random baseline’ set-up had the lowest number of recommendations (ranging from six drugs with k=1 to one drug with k=100).

### 3.2 Beyond sensitivity: the challenge of targeting resistance in drug recommendation systems

After examining the model-free baselines, we tested their performance against the Siamese network and ResMLP models by producing ranked recommendations. The recommendations targeted both sensitivity and resistance. They were evaluated with precision at cutoff *n* and the mean average precision at cutoff *n*. In this context, sensitivity and resistance refer to the pathogen’s response to the effects of a drug, either by being susceptible to its therapeutic action or by having mechanisms to withstand its impact.

Figure 2b illustrates the precision at cut-offs 1–5 for both sensitivity and resistance. For the ‘baseline species’ set-up, we also consider the additional option of setting *k* to the maximum number of samples available in the training set that correspond to the same species that the sample investigated in the testing set (baseline species option 2). With this approach, *k* changes from one test sample to another. In the sensitivity recommendation setting, the performance of the ‘random baseline’ is again the lowest, with the other methods yielding comparable performance. The ‘spectrum similarity’ approach already achieves a very high mean precision (0.97).

Overall, integrating drug fingerprints in the models produced results similar to those from the baseline species approaches for the recommendation task. A limitation of the approaches based on the top-*k* neighbours (including the ‘baseline species’ set-up) is that we cannot evaluate the precision for drugs not tested in the top-*k* neighbours. The precision decreases when the drug cut-off increases for all methods in the resistance setting. This is likely due to the low numbers of drugs to which samples in the testing set are resistant (3.2 on average versus 12.3 for sensitivity).

In general, the precision at cut-offs 1–5 of the drug sensitivity recommendation (dashed lines) is overall higher than the corresponding precision for the drug resistance recommendation. However, this could be due to the metric used to evaluate the recommendation system. Indeed, the resistance precision at cut-off *n* may decrease due to the test sample being resistant to very few drugs.

To address this, we also evaluated the truncated version of precision at cut-off *n* (Supplementary Fig. S12), confirming that resistance is more challenging than sensitivity as a recommendation target.

Finally, we determined the mean average precision at cutoff *n*, which considers not only the number of correct predictions but also the associated ranking (Fig. 2c). For the identification of the sensitivity, the ‘random baseline’ and the ‘random baseline option 1’ still lead to the lowest performance. The other methods give very close results. However, for the identification of the resistance, from cutoff n=2, the ‘ResMLP’ model performs better than all the other approaches. Hence, while most approaches are able to recommend a sufficient number of sensitive drugs, the ‘ResMLP’ model demonstrates greater consistency in identifying the most resistant drugs, leading to the highest mAP@n overall. This result highlights the value of using the ‘ResMLP’ model and, more generally, the inclusion of the drug chemical features in the resistance prediction task. Overall, Fig. 2b and c and Supplementary Fig. S12 show that precise drug recommendation offers promising opportunities but also highlights the complexity of the task. These analyses motivate further research on the methodological developments of MALDI-TOF mass spectra and drug molecular fingerprinting for antimicrobial recommendation.

### 3.3 Joint modelling of chemical and proteomics information outperforms single-species single-drug classifiers

To evaluate the effectiveness of joint multimodal learning, we compared our deep learning model to the more restricted ML-based approaches proposed in Weis *et al.* (2022), where a single model is trained for each drug/species combination.

We selected the same drug–species combinations described in the article and adopted a 5-fold validation scheme to estimate the test performance using AUROC and AUPRC as metrics. For each combination, the corresponding samples are held out from the DRIAMS A set and split into 5 folds. A ResMLP is trained on all the remaining DRIAMS A samples, using the same configuration adopted for the resistance prediction experiments 3.4, to obtain a pretrained network. Finally, for each of the five test splits, the remaining four splits for the target combination are used to fine-tune the model for a further 20 epochs with a reduced learning rate before outputting the predictions for the test fold.

**Figure 3.**
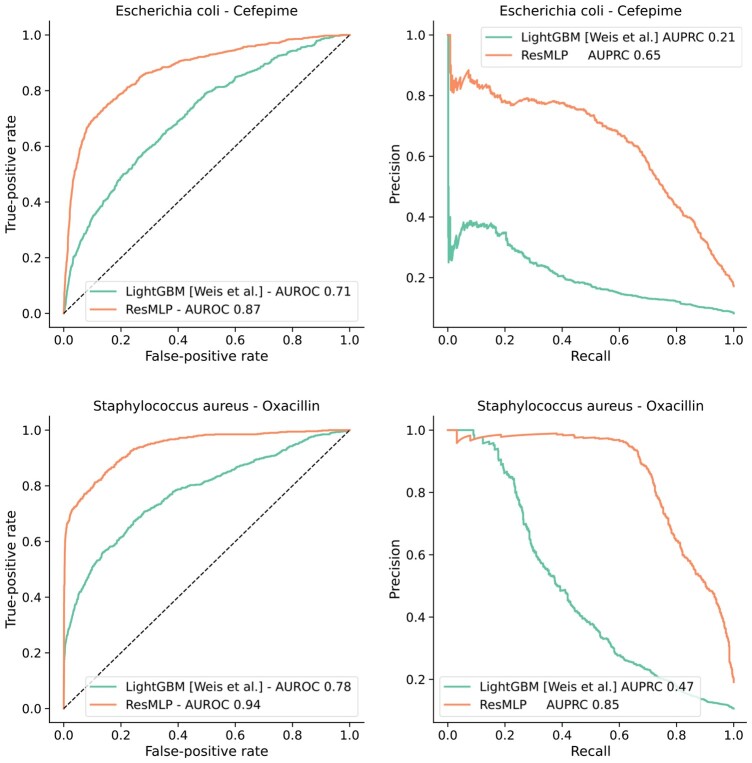
Comparison with the best-performing model from the SOTA reported in Weis *et al.* (2022) for the drug–species combinations for the ResMLP model shows the largest improvements. The full set of ROC and PR curves and the reproduction of Fig. 2 from Weis *et al.* (2022) using our model can be found in the Supplementary Material.

This procedure involves a considerably larger amount of data than the models from Weis *et al.* (2022), which were trained using only samples for the target combination. The results in Fig. 3 highlight how our multi-drug model significantly outperforms the baseline models on most prediction tasks.

Over the 12 drug–species pairings featured in Weis *et al.*, ResMLP attains an average increase in AUROC of +0.12(SD=0.09) and AUPRC of +0.25(SD=0.18) when compared to the LightGBM baseline and comparable results against the MLP baseline (+0.12(SD=0.08) and +0.24(SD=0.18), respectively). A Wilcoxon signed-rank test between the metrics for the ResMLP model and the baselines yields *P*-values of .003 (LightGBM) and 0.001 (MLP) for the AUROC comparison and values 0.002 (LightGBM) and 0.001 (MLP) for the AUPRC comparison. With a threshold of 0.05, the improvements brought by the ResMLP model result as statistically significant, even after adjusting for multiple hypothesis testing with the Bonferroni correction.

Overall, the performance displayed by large-scale deep learning models on AMR datasets offers exciting opportunities to leverage the information in the MALDI-TOF spectra, paving the way for future potential applications in a clinical setting. Moreover, it is important to emphasize that whereas we compare against a classical ML method in this case, we use the best available model from Weis *et al.* (2022), which tested MLP architectures as well. This highlights the benefits of multimodal learning over a larger dataset rather than mere architectural advantages from a larger network. Moreover, we note that previous SOTA results, which we have outperformed, had already demonstrated improvements in retrospective clinical analysis, suggesting these generalized models could have a significant real-world impact.

### 3.4 Deep learning enables accurate predictions of AMR in the i.i.d. setting

The performance of AMR prediction models can vary significantly depending on the data-generating process of the target prediction task.

We performed a set of experiments to analyse the predictive performance of our models in the three data splits described in Section 2. The ‘random’, ‘species-drug zero-shot’, and ‘drugs zero-shot’ splits correspond to the i.i.d. setting, the generalization to novel species–drug combination, and the generalization to new drugs, respectively.

The best results obtained by each model, shown in Table 1 for the dataset DRIAMS B and in Supplementary Table S2 for all collection sites, reveal several interesting aspects of the AMR resistance prediction task. The i.i.d. setting of the ‘random split’ allows the models to produce the best possible results, while the out-of-distribution splits pose a considerable challenge for obtaining accurate predictions.

**Table 1.** Direct AMR prediction results with multi-drug models on the DRIAMS B dataset.^a^

Split type	Model	Cross-validation performance score—mean (SD)		
		AUPRC	Bal. accuracy	MCC
Random	PCA+LR	0.64 (0.02)	0.705 (0.007)	0.51 (0.02)
	Siamese+LR	0.49 (0.01)	0.76 (0.01)	0.53 (0.02)
	Sp-ResMLP	0.35 (0.04)	0.59 (0.03)	0.21 (0.05)
	ResMLP	**0.87 (0.02)**	**0.90 (0.01)**	**0.79 (0.02)**
Species-drug zero-shot	PCA+LR	0.44 (0.04)	0.63 (0.02)	0.30 (0.04)
	Siamese+LR	0.42 (0.01)	0.664 (0.004)	**0.40 (0.01)**
	Sp-ResMLP	0.52 (0.04)	0.62 (0.02)	0.30 (0.04)
	ResMLP	**0.54 (0.04)**	**0.70 (0.02)**	0.39 (0.03)
Drug zero-shot	PCA+LR	0.33 (0.25)	0.57 (0.12)	0.12 (0.16)
	Siamese+LR	0.18 (0.16)	0.52 (0.05)	0.08 (0.14)
	Sp-ResMLP	0.17 (0.16)	0.50 (0.12)	0.01 (0.17)
	ResMLP	**0.47 (0.31)**	**0.71 (0.15)**	**0.35 (0.28)**

a The average metrics are reported together with their SD that is obtained by repeating the analysis over multiple randomization seeds for the ‘Random’ and ‘Species-drug zero-shot’ splits, and for each held-out drug in the ‘Drug zero-shot split’. In bold, we highlighted the best metric across models for a specific split.

The ResMLP model outperformed the other approaches in several prediction settings by significant margins. This model represents the largest of the methods tested, with ∼8.9M trainable parameters in the final configuration adopted, and required a much higher computational cost with training that included up to several hundred epochs of optimization (with a certain amount of variability due to the use of early stopping). This result suggests that the AMR prediction task benefits from using large-scale deep learning models, whose success is predicated on collecting large quantities of data.

The ‘species-drug zero-shot’ split leads to a noticeable degradation in performance for all models except the ablation experiment ‘Sp-ResMLP’, suggesting that training the model on data that captures the interaction between specific pathogenic samples and antimicrobial drugs is crucial to leverage the information contained in the MALDI-TOF spectra.

The ‘drug zero-shot’ prediction task was a difficult challenge for all models, as indicated by the large SDs in the measured metrics (Table 1).

The high variability in performance can be attributed in part to the heterogeneous test splits for this task. Unlike the other two test settings, where the overall class balance from the dataset can be maintained with stratified splits, the class imbalance in the test set can vary significantly depending on the held-out target drug (see Supplementary Fig. S6). Additionally, we speculated that the test performance for a held-out drug could depend on its similarity to the remaining compounds in the training set. However, further analysis in this direction failed to reveal any such correlation (see Supplementary Fig. S11).

The full set of plots showcasing the test AUPRC in the drug zero-shot split is available in Supplementary Fig. S8.

### 3.5 Ablation experiments and feature importance show the value of combining MALDI-TOF spectra with chemical features

We evaluated various configurations and design options for each model. These included early integration of the MALDI-TOF and chemical fingerprint, dimensionality reduction with a single PCA projection, and the use of different chemical fingerprints and classifiers for the PCA and Siamese methods. However, none of these design choices yielded results that surpassed those of the deep learning-based ResMLP model.

To determine the value added by the MALDI-TOF spectra in predicting AMR compared to considering only the species of the bacterial samples, we trained a ResMLP model by replacing the input of the MALDI-TOF spectra with a simple 1-hot encoding of the species. The results, as shown in Table 1 under the label ‘Sp-ResMLP’, indicated a significant decline in performance in most test scenarios.

During our experimentation, we tested the use of different molecular fingerprinting methods. The use of feature importance analysis revealed the value of using chemical fingerprinting methods. However, no specific fingerprint class emerged as consistently superior to the others (Supplementary Table S3 and Fig. S7). Where it is not otherwise specified, we made use of the 1024D Morgan fingerprints (also known as ECFP4), which we chose since it is one of the most popular molecular representations used for small molecule screening, which has demonstrated robust performance in several tasks.

Finally, we utilized SHAP values (Lundberg and Lee 2017) to quantify the contributions of the sets of spectral and chemical features for AMR in a ResMLP model trained using the MACCS chemical fingerprints. Analysing the feature importance grouped by data type (Supplementary Fig. S9) and the most important features (Supplementary Fig. S10) showed that both the spectrum and the fingerprint features played an important role in the final prediction, corroborating our design choices.

**Figure 4.**
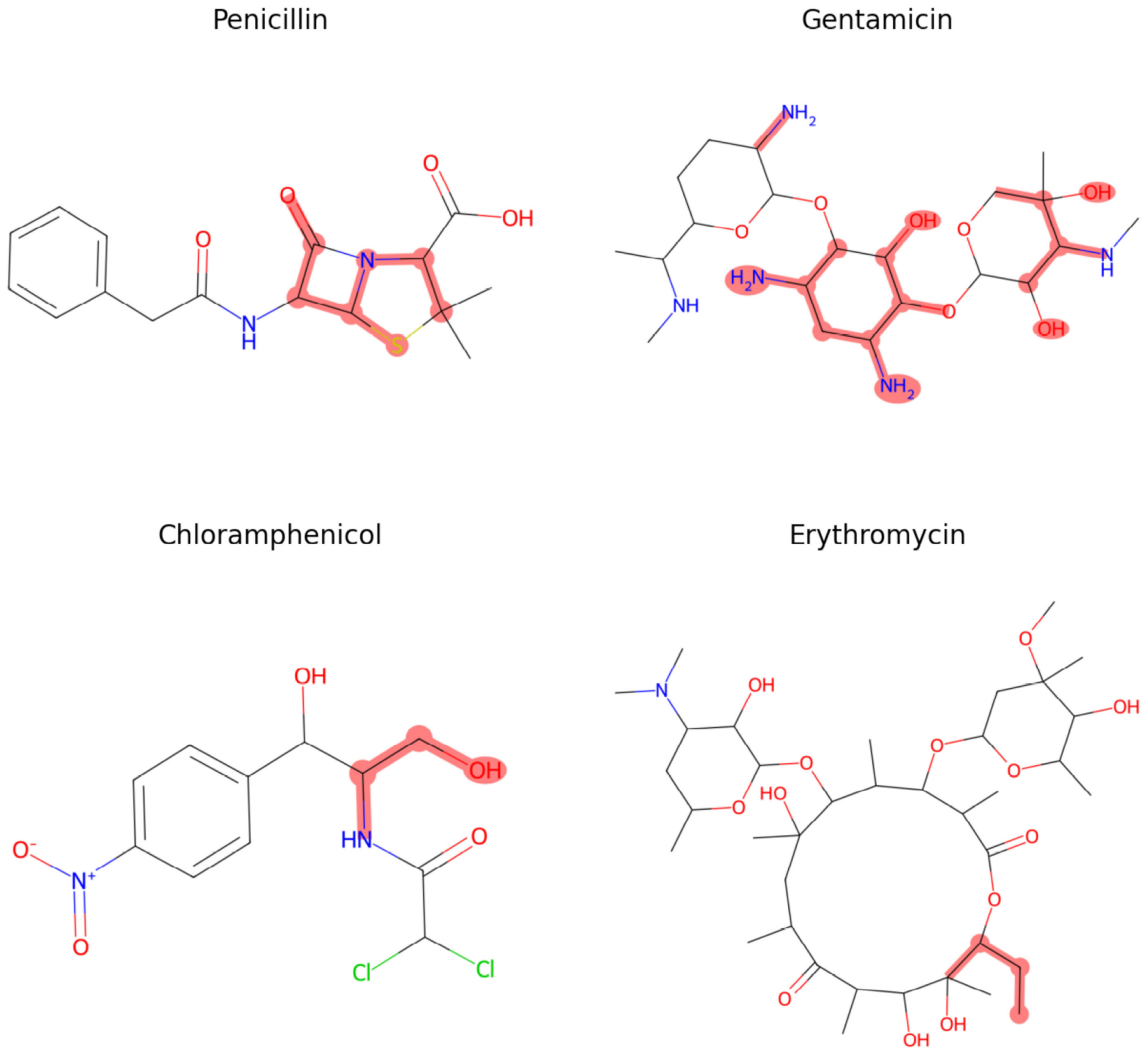
Chemical structures of representative drugs in four common antimicrobial families, with top SHAP substructures highlighted using a wider overlayed area. The highest-scoring features are associated with known mechanisms of action.

Mapping back the highlighted features to the input MACCS fingerprints uncovered intriguing patterns related to well-known AMR mechanisms (Leclercq 2002, Worthington and Melander 2013, Garneau-Tsodikova and Labby 2016). Specifically, our findings demonstrated that for beta-lactam antimicrobials, the beta-lactam ring was a critical feature, especially in penicillins. The top features of aminoglycoside antimicrobials included amine or alcohol groups from sugar rings. Chloramphenicol and macrolide antimicrobials also displayed significant chemical features that align with known resistance mechanisms. These insights may inform the design of novel antimicrobials with improved resistance profiles. A visual representation of these findings can be seen in Fig. 4, where antimicrobial structures are displayed with highlighted atoms corresponding to the discussed chemical features.

## 4 Discussion

This study explored the integration of chemical and proteomics data to predict AMR outcomes. By employing deep learning models, we examined the benefits of combining patient MALDI-TOF mass spectra with chemical fingerprints, which can be collected in a time-sensitive manner, making it highly relevant for various clinical applications. Our results indicate that combining information from multiple drugs and species outperforms existing methods and demonstrates the potential of transferring chemical knowledge to improve AMR predictions. Moreover, we showed the generalizability of our approach by incorporating drug information and evaluating its effectiveness on unseen compounds.

The use of deep learning in AMR prediction is not new. In contrast to other methods that require genetic sequencing (Arango-Argoty *et al.* 2018), we rely on publicly available information on drug structure and mass spectrometry data, which is already a routine for species identification, preceding cell culture to identify the best antimicrobial treatment (Weis *et al.* 2022). We expect that this work will offer new insights to the AMR community in the direction of unifying knowledge and eventually deciphering the relationship between pathogen composition and chemical features of treatments.

Exploring further the idea of reasoning over chemical space for AMR prediction, we proposed recommendation systems that can robustly predict drugs with a high chance of sensitivity or resistance for unseen spectra. By reducing the application of generic, nonspecific medications in most cases, ML models could also help prevent antimicrobial overuse.

Proteomics and genomics have both been used in AMR prediction (Yoon and Jeong 2021, Feucherolles *et al.* 2022, Kim *et al.* 2022, Ren *et al.* 2022). Proteomics, notably MALDI-TOF MS, offers a rapid and cost-effective diagnostic method for infectious diseases in clinical settings. It provides real-time insights into organism responses to antibiotics and functional information, aiding in tracking adaptive responses and discovering resistance mechanisms. Genomics, on the other hand, provides stable DNA data for consistent comparisons and identifies intrinsic resistance mechanisms like gene mutations. Molecular diagnostics like PCR swiftly detect resistance genes, but often target single genes and lack comprehensive insight into nongenetically mediated resistance mechanisms.

Although our primary focus is on the application of MLPs in AMR prediction, there is also potential in multi-label classification approaches (Tsoumakas *et al.* 2010). For instance, ensemble methods (Rokach *et al.* 2014) could offer a promising avenue for further investigation. Moreover, the astonishing progress in graph neural networks (GNNs) (Bongini *et al.* 2023) makes it attractive to represent compounds as attribute-rich networks, and representation learning attempts have already shown that these approaches can successfully generate molecules with specific properties (Lee and Min 2022). A follow-up in this direction could help increase performance even further by representing drugs more efficiently. As data collection efforts grow across multiple sites worldwide, the prospect of training large-scale representation learning models, akin to foundation models (Bommasani *et al.* 2021), for AMR prediction appears increasingly attainable. Additionally, evaluating the cross-site generalizability of our models is paramount to ensure the robustness and applicability of our findings across diverse healthcare settings, ultimately enhancing the potential impact of our research.

Nonetheless, our approach’s integration of previous species-drug-specific models represents a strong advantage over the previous SOTA. It constitutes a step towards building technologies that can leverage as much information as possible from different relevant modalities. This advancement holds the promise of significantly enhancing patient care through more precise and adaptable predictive tools.

## Supplementary Material

btad717_Supplementary_Data

## Data Availability

No new data was generated or analysed in support of this research. The DRIAMS dataset is publicly available online (https://datadryad.org/stash/dataset and https://doi.org/10.5061/dryad.bzkh1899q). The code used to produce the results presented in this article is available at https://github.com/BorgwardtLab/MultimodalAMR.

## References

[btad717-B1] Altschul SF , Gish W, Miller W et al Basic local alignment search tool. J Mol Biol 1990;215:403–10. 2231712 10.1016/S0022-2836(05)80360-2

[btad717-B2] Arango-Argoty G , Garner E, Pruden A et al DeepARG: a deep learning approach for predicting antibiotic resistance genes from metagenomic data. Microbiome 2018;6:1–15. 29391044 10.1186/s40168-018-0401-z PMC5796597

[btad717-B3] Arena F , Giani T, Pollini S et al Molecular antibiogram in diagnostic clinical microbiology: advantages and challenges. *Future Microbiol* 2017;12:361–4. 10.2217/fmb-2017-0019. 28361554

[btad717-B4] Bajorath J. Selected concepts and investigations in compound classification, molecular descriptor analysis, and virtual screening. J Chem Inf Comput Sci 2001;41:233–45. 11277704 10.1021/ci0001482

[btad717-B5] Baptista D , Ferreira PG, Rocha M. Deep learning for drug response prediction in cancer. Brief Bioinform 2021;22:360–79. 31950132 10.1093/bib/bbz171

[btad717-B6] Barlam TF , Cosgrove SE, Abbo LM et al Implementing an antibiotic stewardship program: guidelines by the Infectious Diseases Society of America and the Society for Healthcare Epidemiology of America. Clin Infect Dis 2016;62:e51–77. 27080992 10.1093/cid/ciw118 PMC5006285

[btad717-B7] Benkova M , Soukup O, Marek J. Antimicrobial susceptibility testing: currently used methods and devices and the near future in clinical practice. J Appl Microbiol 2020;129:806–22. 32418295 10.1111/jam.14704

[btad717-B8] Bommasani R , Hudson DA, Adeli E et al On the opportunities and risks of foundation models. arXiv, arXiv:2108.07258, 2021, preprint: not peer reviewed. 10.48550/arXiv.2108.07258.

[btad717-B9] Bongini P , Pancino N, Scarselli F et al BioGNN: how graph neural networks can solve biological problems. *Artificial Intelligence and Machine Learning for Healthcare* 2022. Springer Cham. Vol. 1: 211–231.

[btad717-B10] Bookstaver PB , Nimmich EB, Smith TJ et al Cumulative effect of an antimicrobial stewardship and rapid diagnostic testing bundle on early streamlining of antimicrobial therapy in gram-negative bloodstream infections. Antimicrob Agents Chemother 2017;61:e00189–17. 28630187 10.1128/AAC.00189-17 PMC5571292

[btad717-B11] Chicco D. Siamese neural networks: an overview. Artif Neural Netw 2021;2190:73–94. 10.1007/978-1-0716-0826-5_3 32804361

[btad717-B12] Chiu Y-C , Chen H-IH, Zhang T et al Predicting drug response of tumors from integrated genomic profiles by deep neural networks. BMC Med Genomics 2019;12:143–55. 30704458 10.1186/s12920-018-0460-9 PMC6357352

[btad717-B13] Corbin CK , Sung L, Chattopadhyay A et al Personalized antibiograms for machine learning driven antibiotic selection. Commun Med (Lond) 2022;2:38–14. 35603264 10.1038/s43856-022-00094-8 PMC9053259

[btad717-B14] Costello JC , Heiser LM, Georgii E et al; NCI DREAM Community. A community effort to assess and improve drug sensitivity prediction algorithms. Nat Biotechnol 2014;32:1202–12. 24880487 10.1038/nbt.2877 PMC4547623

[btad717-B15] David L , Thakkar A, Mercado R et al Molecular representations in AI-driven drug discovery: a review and practical guide. J Cheminform 2020;12:56–22. 33431035 10.1186/s13321-020-00460-5 PMC7495975

[btad717-B16] De Carolis E , Vella A, Vaccaro L et al Application of MALDI-TOF mass spectrometry in clinical diagnostic microbiology. J Infect Dev Ctries 2014;8:1081–8. 25212071 10.3855/jidc.3623

[btad717-B17] Durant JL , Leland BA, Henry DR et al Reoptimization of MDL keys for use in drug discovery. J Chem Inf Comput Sci 2002;42:1273–80. 12444722 10.1021/ci010132r

[btad717-B18] Feldgarden M , Brover V, Haft DH et al Validating the AMRFinder tool and resistance gene database by using antimicrobial resistance genotype-phenotype correlations in a collection of isolates. Antimicrob Agents Chemother 2019;63:10–1128. 10.1128/AAC.00483-19 PMC6811410 31427293

[btad717-B19] Feucherolles M , Nennig M, Becker SL et al Combination of MALDI-TOF mass spectrometry and machine learning for rapid antimicrobial resistance screening: the case of *Campylobacter* spp. Front Microbiol 2022;12:804484. 10.3389/fmicb.2021.804484 PMC8894766 35250909

[btad717-B21] Garneau-Tsodikova S , Labby KJ. Mechanisms of resistance to aminoglycoside antibiotics: overview and perspectives. Medchemcomm 2016;7:11–27. 10.1039/c5md00344j. 26877861 PMC4752126

[btad717-B22] Gönen M , Margolin AA. Drug susceptibility prediction against a panel of drugs using kernelized Bayesian multitask learning. Bioinformatics 2014;30:i556–63. 25161247 10.1093/bioinformatics/btu464 PMC4147917

[btad717-B23] Goodswen SJ , Barratt JLN, Kennedy PJ et al Machine learning and applications in microbiology. FEMS Microbiol Rev 2021;45:fuab015. 33724378 10.1093/femsre/fuab015 PMC8498514

[btad717-B24] Han SS , Jeong YS, Choi SK. Current scenario and challenges in the direct identification of microorganisms using MALDI TOF MS. Microorganisms 2021;9:1917. 34576812 10.3390/microorganisms9091917 PMC8466008

[btad717-B25] He X , Folkman L, Borgwardt K. Kernelized rank learning for personalized drug recommendation. Bioinformatics 2018;34:2808–16. 29528376 10.1093/bioinformatics/bty132 PMC6084606

[btad717-B26] Jia B , Raphenya AR, Alcock B et al CARD 2017: expansion and model-centric curation of the comprehensive antibiotic resistance database. Nucleic Acids Res 2016;45:D566–73. 27789705 10.1093/nar/gkw1004 PMC5210516

[btad717-B27] Kim JI , Maguire F, Tsang KK et al Machine learning for antimicrobial resistance prediction: current practice, limitations, and clinical perspective. Clin Microbiol Rev 2022;35:e00179. 35612324 10.1128/cmr.00179-21 PMC9491192

[btad717-B28] Landrum G , Tosco P, Kelley B et al *rdkit/rdkit: 2021_09_4 (q3 2021) release*. Zenodo 2022.

[btad717-B29] Leclercq R. Mechanisms of resistance to macrolides and lincosamides: nature of the resistance elements and their clinical implications. Clin Infect Dis 2002;34:482–92. 10.1086/324626. 11797175

[btad717-B30] Lee M , Min K. MGCVAE: multi-objective inverse design via molecular graph conditional variational autoencoder. J Chem Inf Model 2022;62:2943–50. 10.1021/acs.jcim.2c00487. 35666276

[btad717-B31] Li Y , Xu Z, Han W et al HMD-ARG: hierarchical multi-task deep learning for annotating antibiotic resistance genes. Microbiome 2021;9:1–12. 33557954 10.1186/s40168-021-01002-3 PMC7871585

[btad717-B32] Lundberg SM , Lee SI. A unified approach to interpreting model predictions. Adv Neural Inform Process Syst 2017;30:4765–4774.

[btad717-B33] Mangioni D , Viaggi B, Giani T et al Diagnostic stewardship for sepsis: the need for risk stratification to triage patients for fast microbiology workflows. *Future Microbiology* 2019;14.3:169–174. 10.2217/fmb-2018-0329 30628478

[btad717-B34] Morgan HL. The generation of a unique machine description for chemical structures – a technique developed at chemical abstracts service. J Chem Doc 1965;5:107–13.

[btad717-B35] Murray CJL , Ikuta KS, Sharara F et al Global burden of bacterial antimicrobial resistance in 2019: a systematic analysis. Lancet 2022;399:629–55. 35065702 10.1016/S0140-6736(21)02724-0 PMC8841637

[btad717-B36] Ren Y , Chakraborty T, Doijad S et al Multi-label classification for multi-drug resistance prediction of *Escherichia coli*. Comput Struct Biotechnol J 2022;20:1264–70. 35317240 10.1016/j.csbj.2022.03.007 PMC8918850

[btad717-B37] Rokach L , Schclar A, Itach E. Ensemble methods for multi-label classification. Exp Syst Appl 2014;41:7507–23.

[btad717-B38] Sabença C , Sousa TD, Oliveira S et al Next-generation sequencing and MALDI mass spectrometry in the study of multiresistant processed meat vancomycin-resistant enterococci (VRE). Biology (Basel) 2020;9:89. 32349310 10.3390/biology9050089 PMC7284646

[btad717-B39] Sogawa K , Watanabe M, Ishige T et al Rapid discrimination between methicillin-sensitive and methicillin-resistant *Staphylococcus aureus* using MALDI-TOF mass spectrometry. Biocontrol Sci 2017;22:163–9. 28954959 10.4265/bio.22.163

[btad717-B40] Sousa T D , Viala D, Théron L et al Putative protein biomarkers of *Escherichia coli* antibiotic multiresistance identified by MALDI mass spectrometry. Biology (Basel) 2020;9:56. 32204308 10.3390/biology9030056 PMC7150737

[btad717-B41] Swain M. PubChemPy Documentation. Release 1.0.4. 2014.

[btad717-B42] Szegedy C , Ioffe S, Vanhoucke V et al Inception-v4, Inception-ResNet and the impact of residual connections on learning. In: *Proceedings of the AAAI Conference on Artificial Intelligence*, San Francisco 2017. AAAI Press. Vol. 31.

[btad717-B43] Tang W , Ranganathan N, Shahrezaei V et al MALDI-TOF mass spectrometry on intact bacteria combined with a refined analysis framework allows accurate classification of MSSA and MRSA. PLoS One 2019;14:e0218951. 31247021 10.1371/journal.pone.0218951 PMC6597085

[btad717-B44] Tsoumakas G , Katakis I, Vlahavas I. Mining multi-label data. In: *Data Mining and Knowledge Discovery Handbook*. 2010. Springer New York, New York. 667–85.

[btad717-B45] Wang H-Y , Chen C-H, Lee T-Y et al Rapid detection of heterogeneous vancomycin-intermediate *Staphylococcus aureus* based on matrix-assisted laser desorption ionization time-of-flight: using a machine learning approach and unbiased validation. Front Microbiol 2018;9:2393. 30364336 10.3389/fmicb.2018.02393 PMC6193097

[btad717-B46] Wang Y , Bryant SH, Cheng T et al PubChem BioAssay: 2017 update. Nucleic Acids Res 2017;45:D955–63. 27899599 10.1093/nar/gkw1118 PMC5210581

[btad717-B47] Weis C , Cuénod A, Rieck B et al DRIAMS: database of resistance information on antimicrobials and MALDI-TOF mass spectra. Dryad 2021.

[btad717-B48] Weis C , Cuénod A, Rieck B et al Direct antimicrobial resistance prediction from clinical MALDI-TOF mass spectra using machine learning. Nat Med 2022;28:164–74. 10.1038/s41591-021-01619-9. 35013613

[btad717-B49] Weis CV , Jutzeler CR, Borgwardt K. Machine learning for microbial identification and antimicrobial susceptibility testing on MALDI-TOF mass spectra: a systematic review. Clin Microbiol Infect 2020;26:1310–7. 32217160 10.1016/j.cmi.2020.03.014

[btad717-B50] Willett P , Barnard JM, Downs GM. Chemical similarity searching. J Chem Inf Comput Sci 1998;38:983–96.

[btad717-B51] Worthington RJ , Melander C. Overcoming resistance to β-lactam antibiotics. J Organ Chem 2013;78:4207–13. 10.1021/jo400236f. PMC3644377 23530949

[btad717-B52] Yin X , Jiang X-T, Chai B et al ARGs-OAP v2. 0 with an expanded SARG database and hidden Markov models for enhancement characterization and quantification of antibiotic resistance genes in environmental metagenomes. Bioinformatics 2018;34:2263–70. 29408954 10.1093/bioinformatics/bty053

[btad717-B53] Yoon EJ , Jeong SH. MALDI-TOF mass spectrometry technology as a tool for the rapid diagnosis of antimicrobial resistance in bacteria. Antibiotics 2021;10:982. 34439032 10.3390/antibiotics10080982 PMC8388893

