# Supplementary material for: Multimodal learning in clinical proteomics: enhancing antimicrobial resistance prediction models with chemical information
Source: Bioinformatics. 2023 Nov 24;39(12):btad717. doi: 10.1093/bioinformatics/btad717 (PMC10724849; doi:10.1093/bioinformatics/btad717)
Supplement: btad717_Supplementary_Data [file btad717_supplementary_data.pdf]

Multimodal learning in clinical proteomics: enhancing antimicrobial resistance models with chemical information

Supplementary Material

Supplementary tables

Table A1. Overview of the DRIAMS dataset

Dataset	Laboratory	Collection period	MALDI-TOF MS	AMR labels	AM drugs
DRIAMS-A	University Hospital Basel	34 months (11/2015–08/2018)	145,341	3 101,660	71
DRIAMS-B	Canton Hospital Basel-Land	6 months (01/2018–06/2018)	6,416	37,453	44
DRIAMS-C	Canton Hospital Aarau	8 months (01/2018–08/2018)	22,500	50,114	56
DRIAMS-D	Viollier	6 months (01/2018–06/2018)	75,813	98,708	52

Table A2. Direct AMR prediction results with multi-drug models. The best average metric is highlighted for each dataset and split.

Dataset	Split type	Model	Cross-validation performance score - mean (SD)		
			**AUPRC**	**Balanced accuracy**	**MCC**
DRIAMS-A	Random	PCA + LR	0.644 (0.003)	0.704 (0.002)	0.491 (0.003)
		Species-ResMLP	0.30 (0.04)	0.53 (0.02)	0.08 (0.05)
		ResMLP	**0.92 (0.01)**	**0.90 (0.01)**	**0.81 (0.01)**
	Species-drug zero-shot	PCA + LR	0.28 (0.02)	0.57 (0.01)	0.16 (0.02)
		Species-ResMLP	0.31 (0.04)	0.54 (0.02)	0.09 (0.03)
		ResMLP	**0.42 (0.03)**	**0.64 (0.02)**	**0.28 (0.03)**
	Drug zero-shot	PCA + LR	0.35 (0.27)	0.55 (0.14)	0.11 (0.25)
		Species-ResMLP	0.34 (0.32)	0.51 (0.07)	0.02 (0.14)
		ResMLP	**0.47 (0.29)**	**0.65 (0.13)**	**0.28 (0.24)**
DRIAMS-B	Random	PCA + LR	0.64 (0.02)	0.705 (0.007)	0.51 (0.02)
		Siamese + LR	0.49 (0.01)	0.76 (0.01)	0.53 (0.02)
		Species-ResMLP	0.35 (0.04)	0.59 (0.03)	0.21 (0.05)
		ResMLP	**0.87 (0.02)**	**0.90 (0.01)**	**0.79 (0.02)**
	Species-drug zero-shot	PCA + LR	0.44 (0.04)	0.63 (0.02)	0.30 (0.04)
		Siamese + LR	0.42 (0.01)	0.664 (0.004)	**0.40 (0.01)**
		Species-ResMLP	0.52 (0.04)	0.62 (0.02)	0.30 (0.04)
		ResMLP	**0.54 (0.04)**	**0.70 (0.02)**	0.39 (0.03)
	Drug zero-shot	PCA + LR	0.33 (0.25)	0.57 (0.12)	0.12 (0.16)
		Siamese + LR	0.18 (0.16)	0.52 (0.05)	0.08 (0.14)
		Species-ResMLP	0.17 (0.16)	0.50 (0.12)	0.01 (0.17)
		ResMLP	**0.47 (0.31)**	**0.71 (0.15)**	**0.35 (0.28)**
DRIAMS-C	Random	PCA + LR	0.62 (0.01)	0.72 (0.01)	0.49 (0.01)
		Species-ResMLP	0.41 (0.11)	0.59 (0.06)	0.17 (0.13)
		ResMLP	**0.92 (0.01)**	**0.89 (0.01)**	**0.81 (0.02)**
	Species-drug zero-shot	PCA + LR	0.39 (0.04)	0.60 (0.02)	0.23 (0.05)
		Species-ResMLP	0.48 (0.05)	0.62 (0.04)	0.28 (0.08)
		ResMLP	**0.55 (0.03)**	**0.69 (0.02)**	**0.39 (0.00)**
	Drug zero-shot	PCA + LR	0.24 (0.29)	0.63 (0.23)	0.03 (0.13)
		Species-ResMLP	0.22 (0.27)	0.47 (0.23)	0.05 (0.26)
		ResMLP	**0.34 (0.35)**	**0.66 (0.25)**	**0.17 (0.28)**
DRIAMS-D	Random	PCA + LR	0.67 (0.01)	0.76 (0.01)	0.60 (0.01)
		Species-ResMLP	0.57 (0.07)	0.71 (0.03)	0.47 (0.08)
		ResMLP	**0.76 (0.01)**	**0.82 (0.01)**	**0.64 (0.01)**
	Species-drug zero-shot	PCA + LR	0.49 (0.06)	0.63 (0.02)	0.36 (0.05)
		Species-ResMLP	**0.67 (0.04)**	**0.72 (0.02)**	**0.51 (0.04)**
		ResMLP	0.63 (0.05)	0.72 (0.03)	0.47 (0.05)
	Drug zero-shot	PCA + LR	0.23 (0.27)	0.52 (0.15)	0.02 (0.04)
		Species-ResMLP	0.18 (0.28)	0.52 (0.16)	0.01 (0.05)
		ResMLP	**0.36 (0.31)**	**0.66 (0.16)**	**0.20 (0.22)**

Table A3. Results of one-way Kruskal-Wallis analysis of variance for comparing the performance of the ResMLP model using the different categories of molecular fingerprints (MACCS, 1024-dimensional Morgan, Pubchem). Using the *random split* setting, 10 train/test splits were performed on each of the 4 DRIAMS collection sites. A ResMLP model was trained using the same configuration as the main section of the classification experiments. The resulting metrics were used to calculate the Kruskal-Wallis H statistic. The resulting p-values indicate that no fingerprint choice leads to significantly different results, using a significance threshold of 0.05.

Metric	Kruskal-Wallis H Statistic	P-Value
MCC	4.296865	0.116667
AUPRC	1.373165	0.503293
Balanced Accuracy	1.367955	0.504606

Supplementary figures for the AMR prediction task

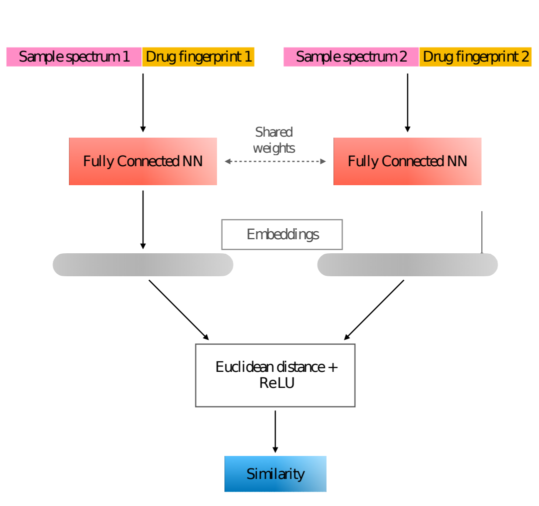

(a) Siamese network

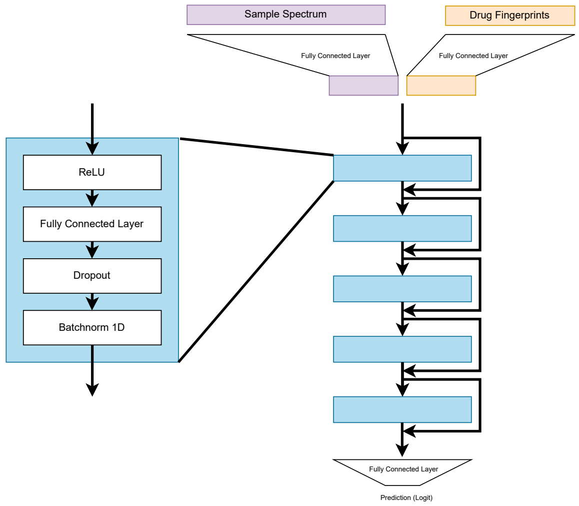

(b) Residual Multi-Layer Perceptron network

Fig. A1: Architecture of the Siamese networks and ResMLP model

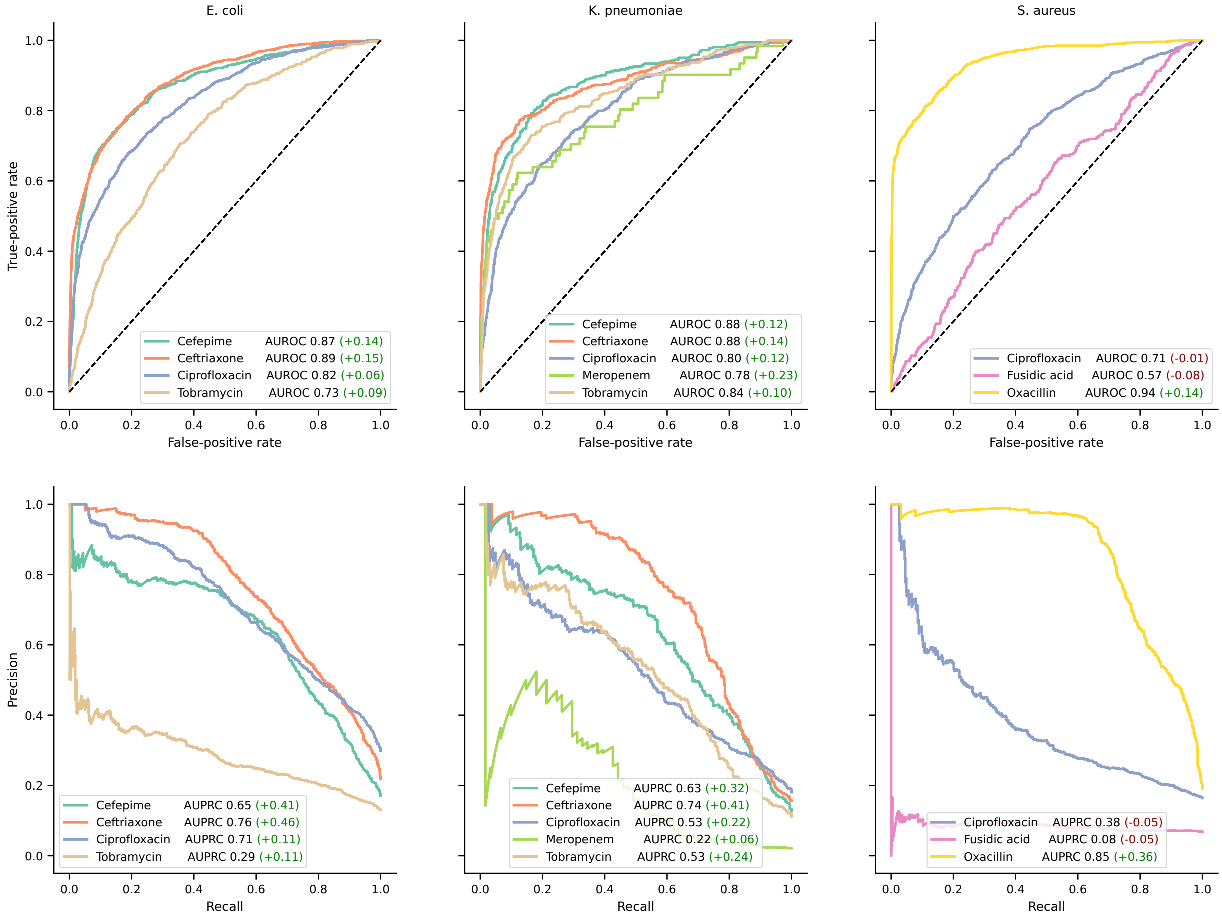

Fig. A2: ROC and PRC curves for selected combinations of species and drugs using the ResMLP model. This figure is a reproduction of Figure 2 from Weis et al. (2022), which showcases how the use of a multi-drug model outperforms the more restricted models presented in their work. In the parentheses next to the AUC measures, the difference compared to the corresponding models in Figure 2 from Weis et al. (2022).

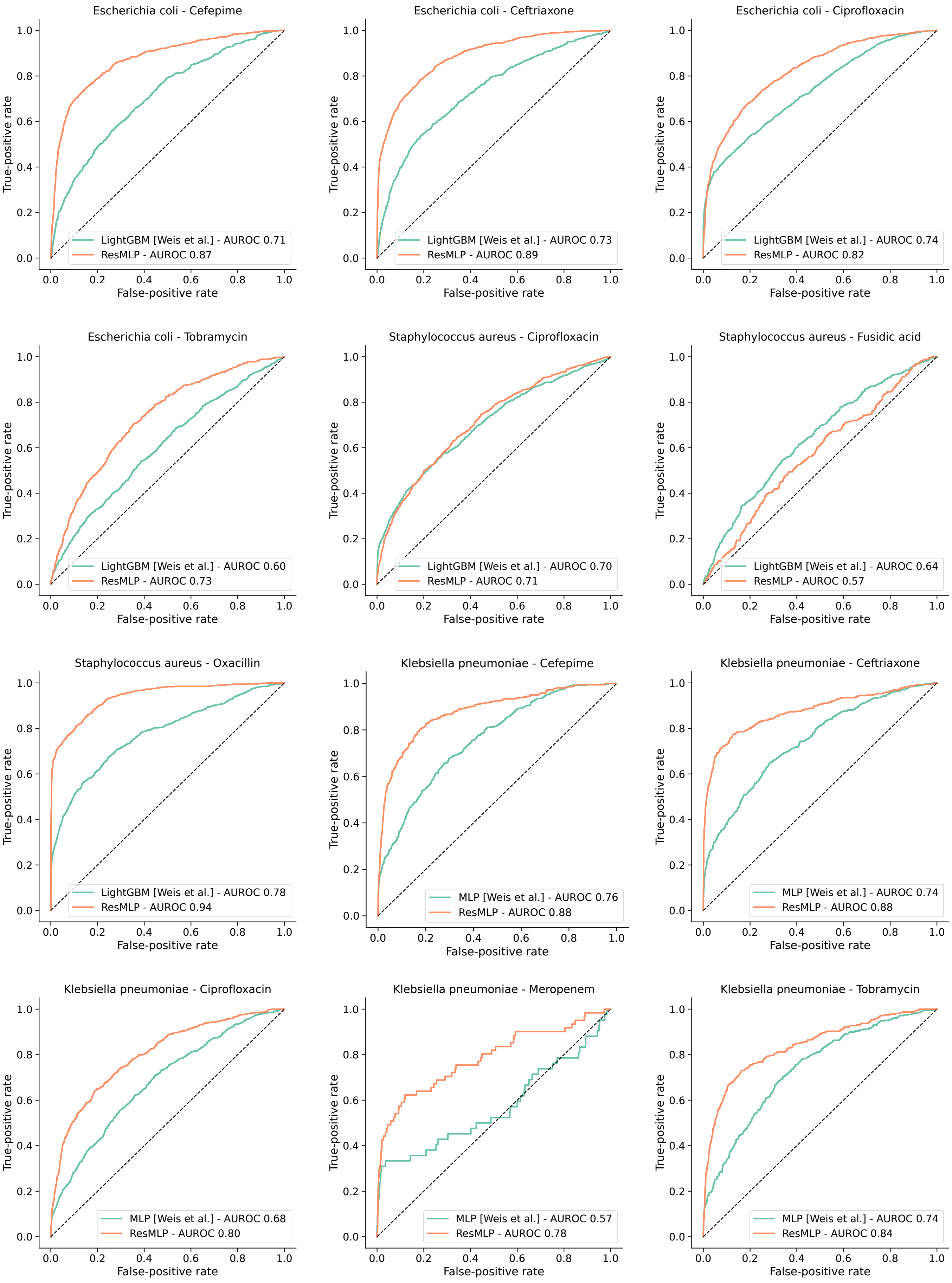

Fig. A3: Full set of Receiver Operating Characteristic curves for the comparison with the models presented in Figure 2 from Weis et al. (2022)

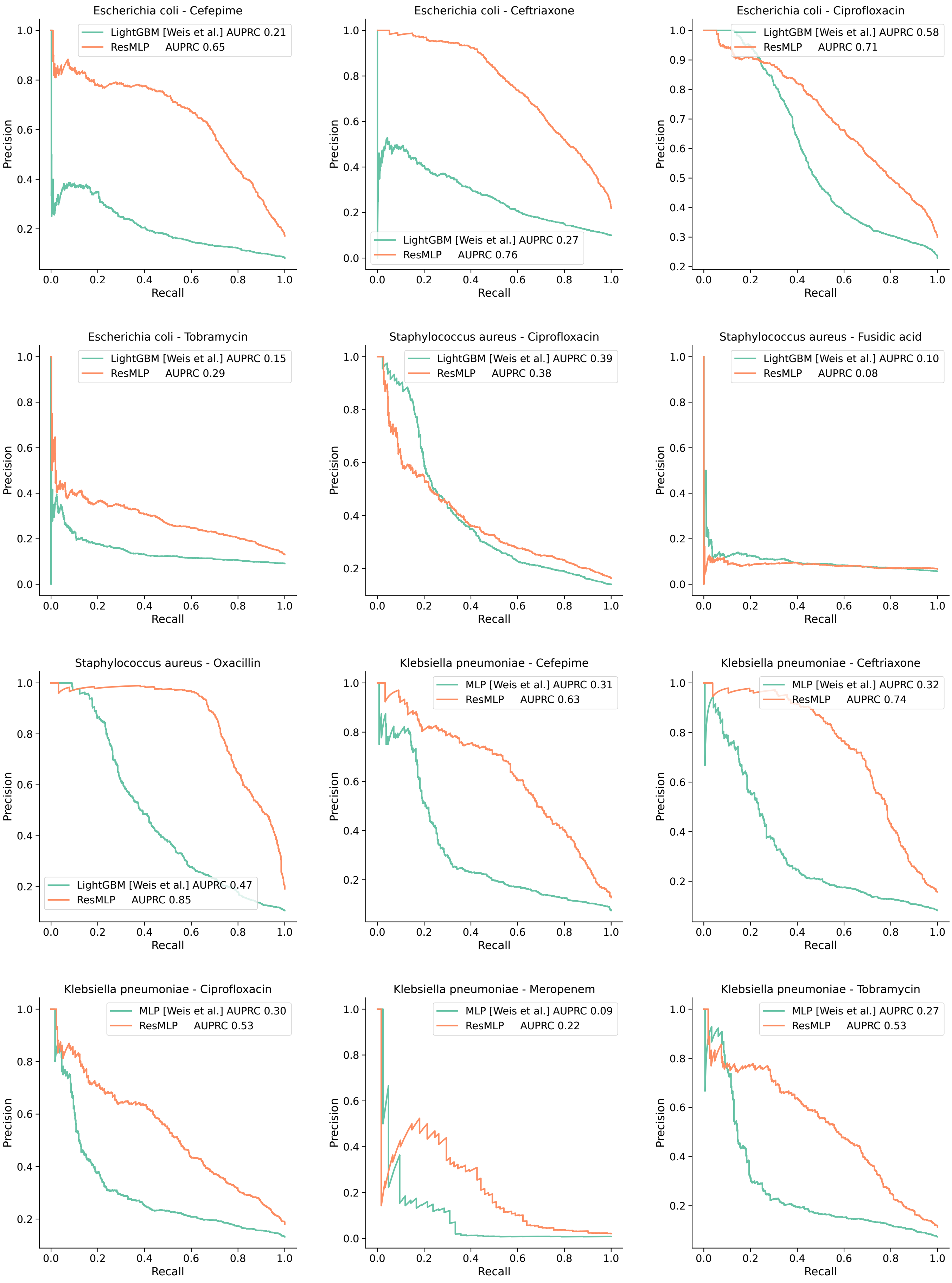

Fig. A4: Full set of Precision-Recall curves for the comparison with the models presented in Figure 2 from Weis et al. (2022)

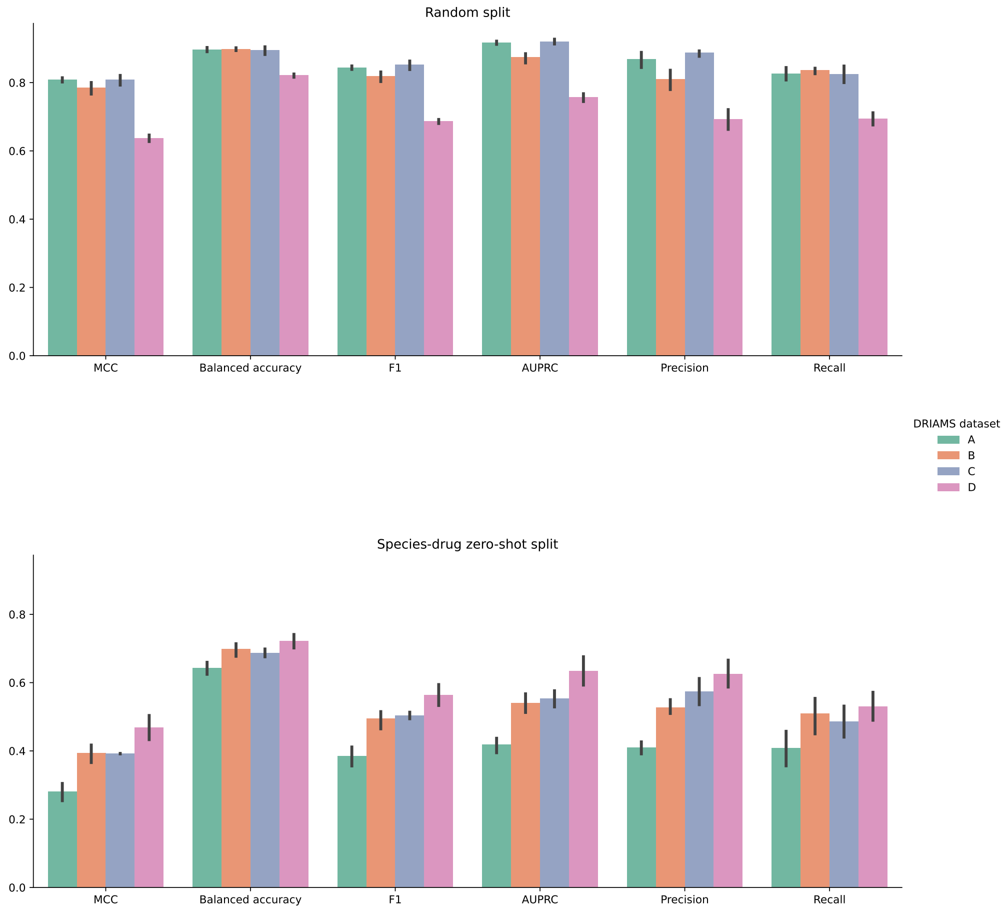

Fig. A5: Comparison of ResMLP predictions on the four DRIAMS datasets for random and species-drug zero-shot data splits. The error bars reported represent a 95% confidence interval.

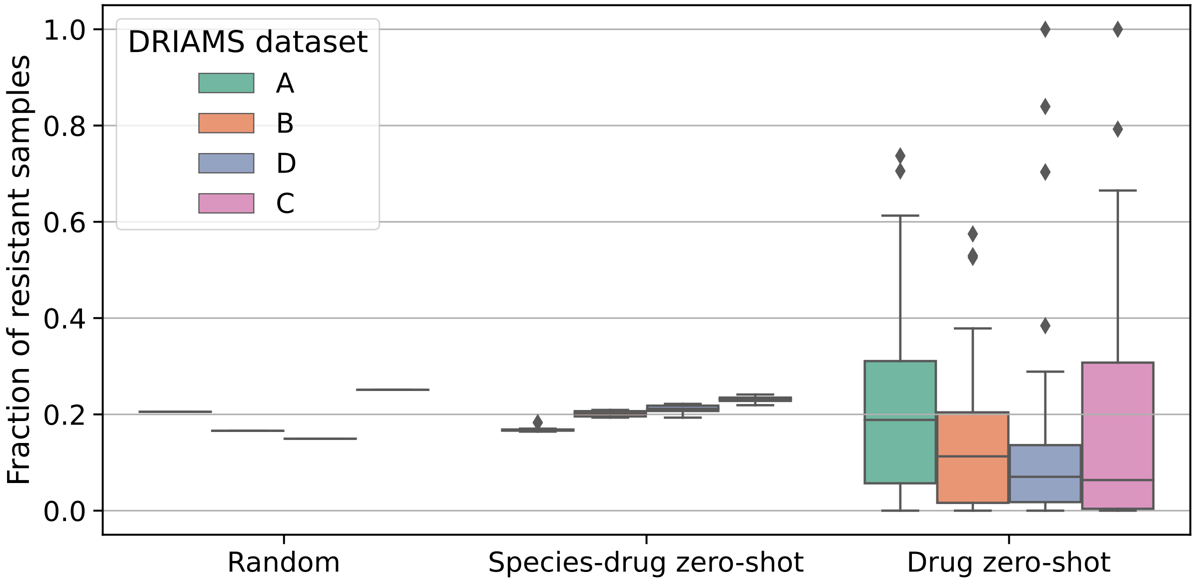

Fig. A6: Fraction of the test samples with a positive label (i.e. displaying resistance to the drug). The random split allows us to sample with stratification to obtain consistent test splits. The species-drug zero-shot split relies on a heuristic to construct test sets that approximately constitute a specific percentage of the total available samples, leading to small fluctuations in the fraction of positive samples in each test split. The drug zero-shot setting, on the other hand, does not allow for any control on the imbalance of the test split. The resulting variability is a challenge for the machine learning models tested to overcome.

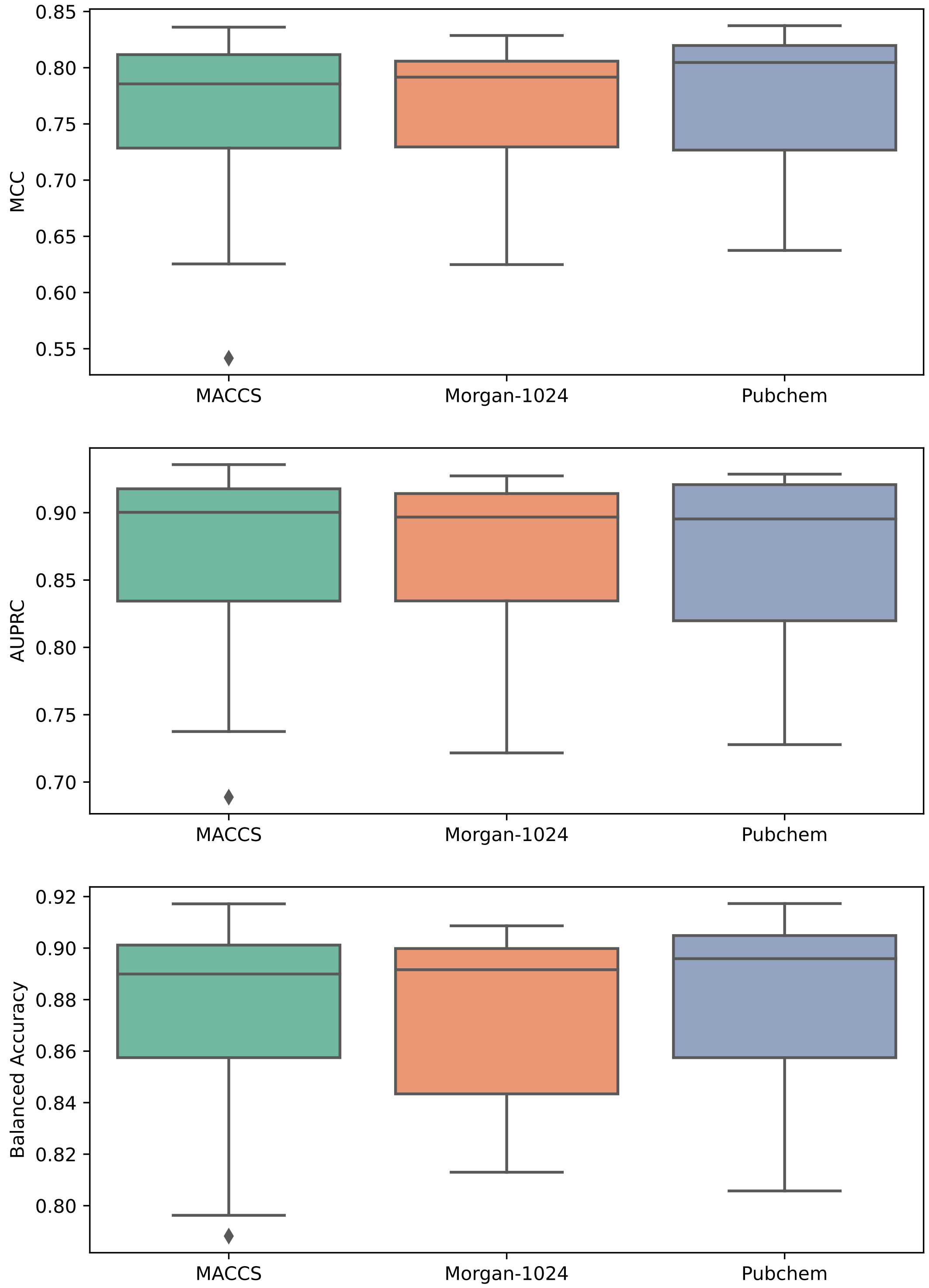

Fig. A7: Comparison of the performance of a ResMLP model trained using the different types of molecular fingerprints. For each DRIAMS collection site, 10 train/test splits are randomly selected, and a ResMLP model is trained using the same configuration presented for the results in the classification performance. The resulting metrics display a small level of variation. An analysis of variance test, however, confirmed that the choice of molecular fingerprint is not statistically significant.

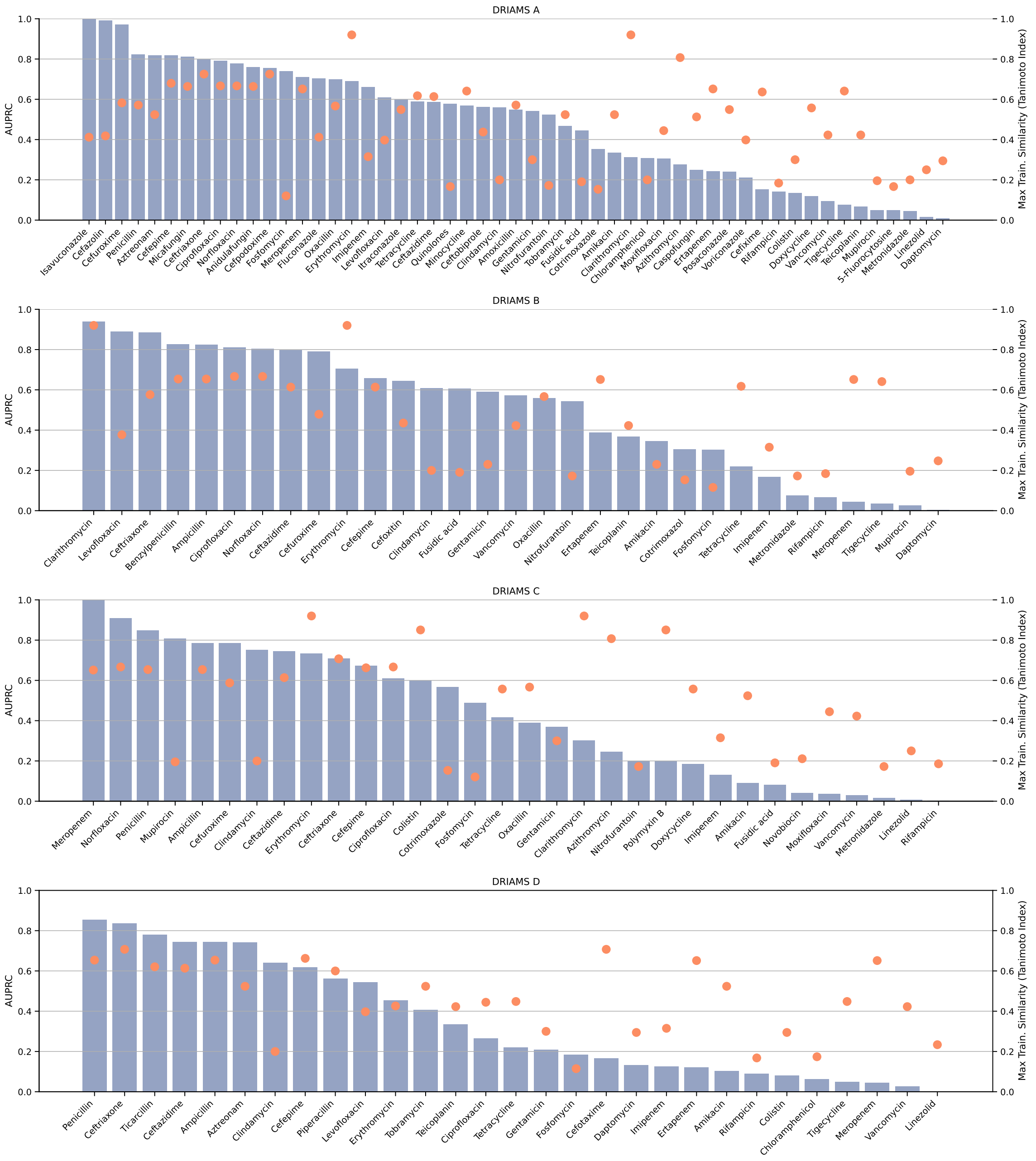

Fig. A8: AUPRC for the predictions in the drug zero-shot task on the four DRIAMS datasets. Drugs for which only one class of response was available have been excluded from the analysis. In orange, we represented as dots the highest Tanimoto index calculated by comparing the MACCS fingerprints of the target drug with the rest of the compounds in the dataset. This measure of similarity does not appear to share a pattern with the generalization performance measured by the AUPRC.

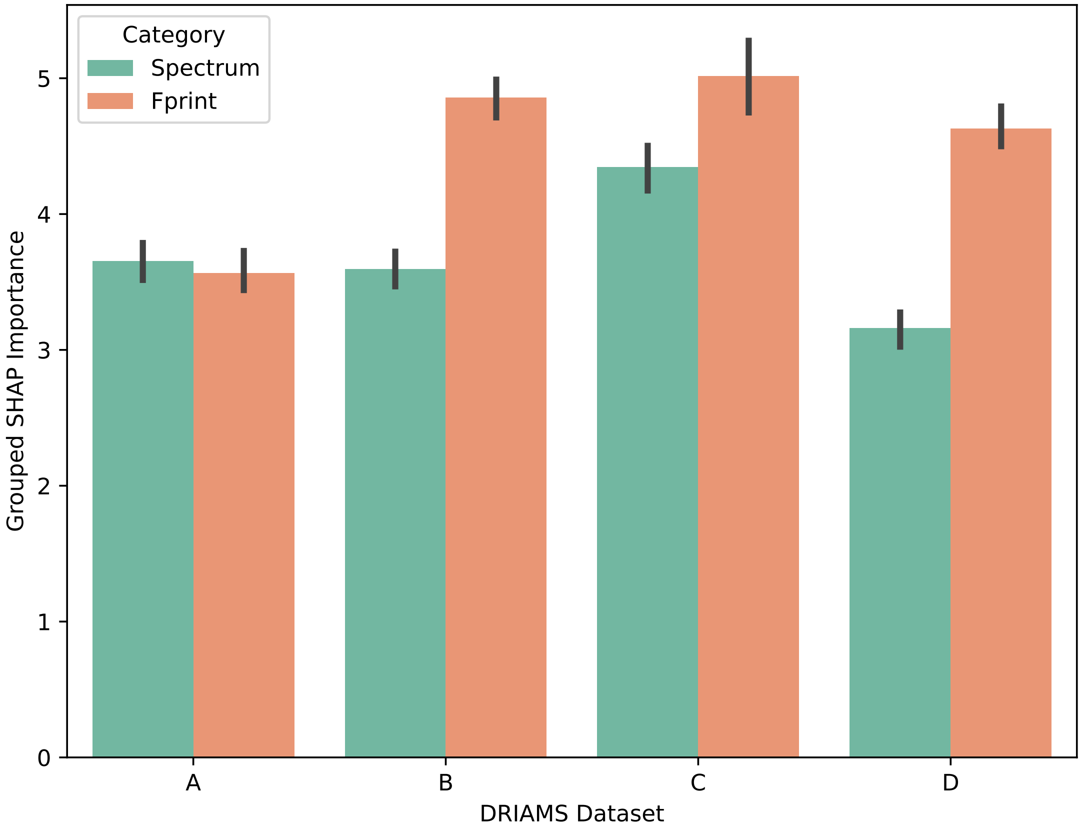

Fig. A9: Grouped SHAP importance for the two sets of features.

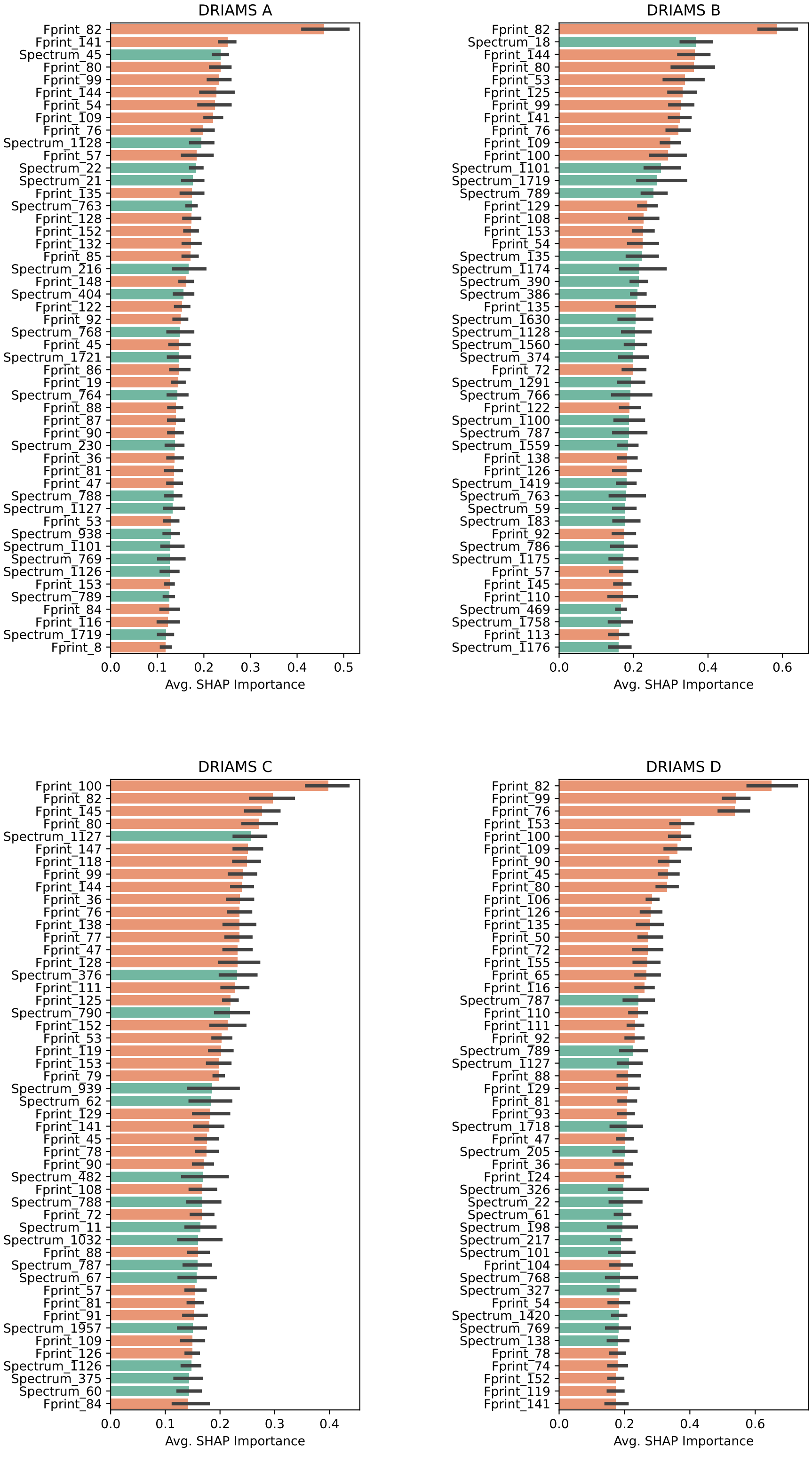

Fig. A10: The 50 most important features for the AMR prediction task for the ResMLP model trained on the MACCS fingerprints, split by DRIAMS dataset. In each case, we clearly see contributions from both the spectrum and the chemical fingerprint.

Supplementary figures for the drug recommendation task

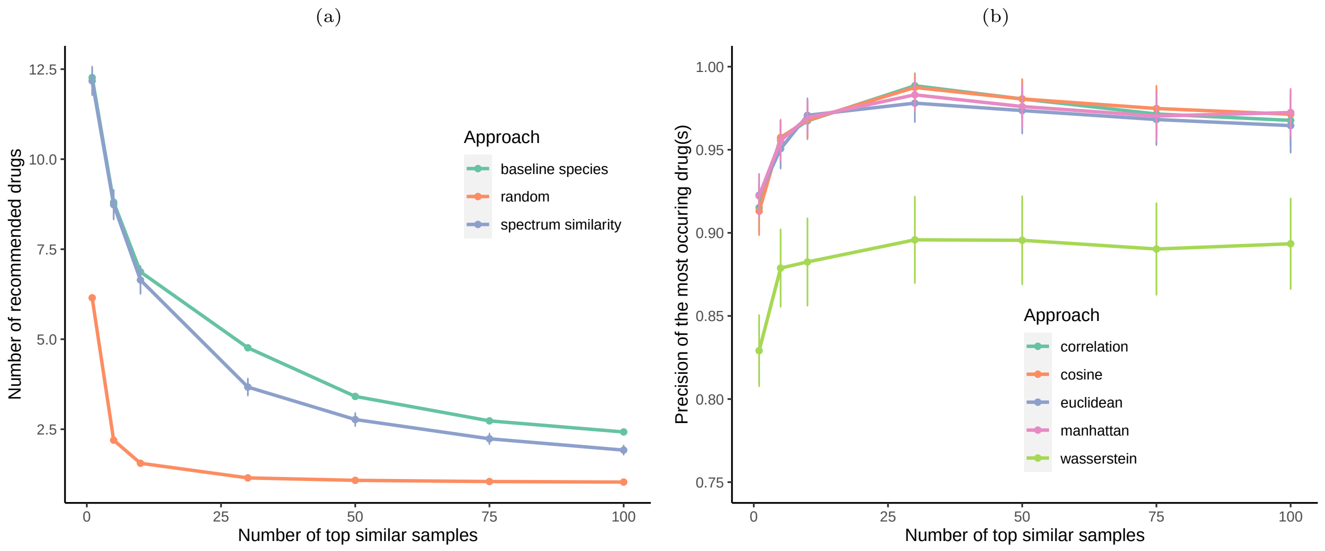

Fig. A11: **(a)** Comparison of the number of drugs recommended with different variations of the approach based on the similarity between spectra. The error bars represent the 95% confidence interval. **(b)** Comparison of performance of different variants of the *spectrum similarity* set-up based on the top k similarity. The y-axis shows the average precision across all individuals in the test set. The error bars represent the 95% confidence interval.

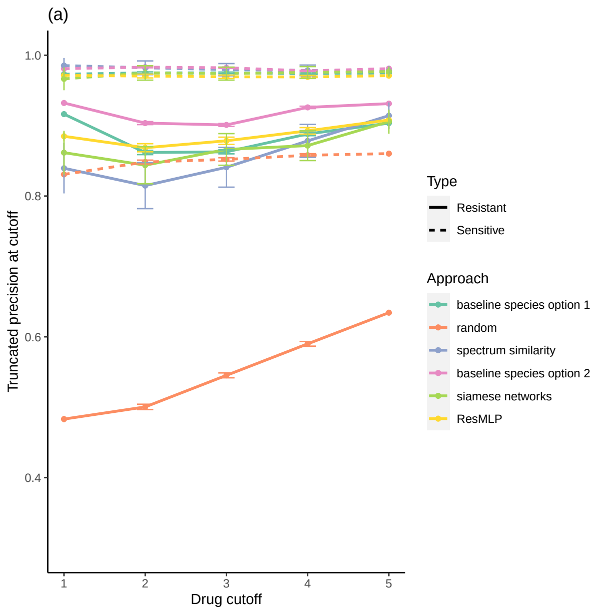

Fig. A12: Truncated precision at cut-offs 1, 2, 3, 4, and 5 for the different recommendation set-ups. For the *random baseline*, *baseline species*, and *spectrum similarity* approaches, the number of top neighbours k is set to 30.

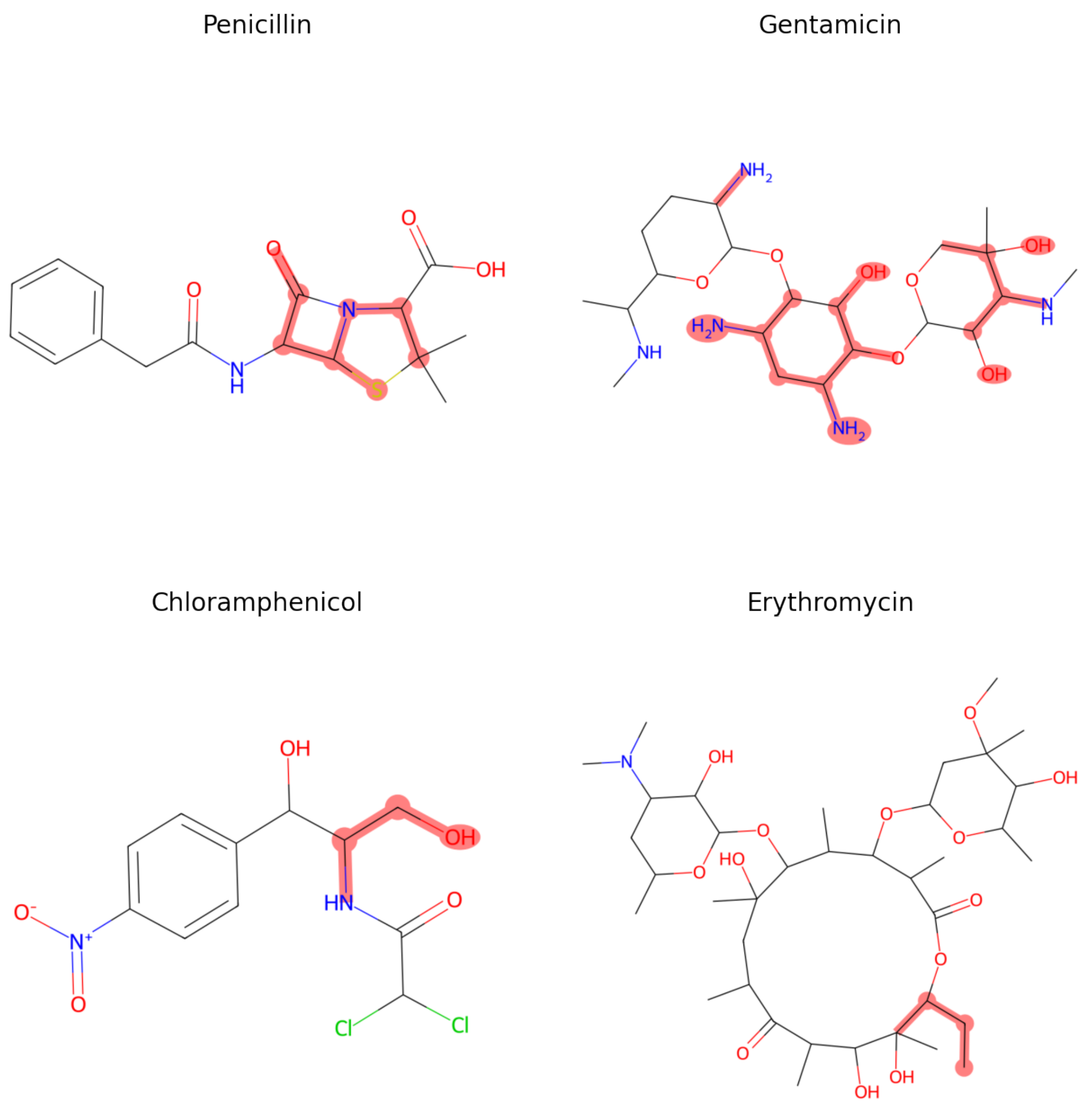

Fig. A13: **Chemical structures of representative drugs in four common antibiotic families**. **Penicillin**: the beta-lactam ring (highlighted in red) is a key structural feature in beta-lactam antibiotics such as Penicillin, where resistance is frequently conferred by beta-lactamase enzymes, which hydrolyze the amide bond, rendering the antibiotic inactive. The beta-lactam ring ranks amongst the first in the SHAP feature importance analyses presented in this paper for most Penicillin antibiotics (such as Amoxicillin, Oxacillin, Ampicillin and Benzylpenicillin). Interestingly, antibiotics that are not susceptible to beta-lactamases (such as Cefepime and Aztreoman) do not follow this trend. **Gentamicin**: is an aminoglycoside antibiotic, as are Amikacin and Tobramycin. Although other resistance mechanisms involving these drugs have been reported, by far the most prevalent involve AG-modifying enzymes that target the glycoside rings and their aglycone components, which matches the top ranked features by the provided SHAP analysis (highlighted in red). **Chloramphenicol** encounters high-level bacterial resistance due to the enzyme chloramphenicol acetyltransferase. This enzyme mediates the transfer of an acetyl group from acetyl CoA to the primary hydroxyl group within the chloramphenicol molecule, which ranks as the top chemical feature in the provided SHAP analysis. **Erythromycin** is a macrolide (such as Azithromycin), a family of antibiotics where drug modification is also the prevalent resistance mechanism in place. Among others, hydrolyzation of the ester group by esterases in particular acts in the ester group next to the atoms highlighted in red (which rank first in the provided feature importance analysis for both mentioned macrolide antibiotics).

Hyperparameters and training configurations

Siamese parameters in all analyses: 512 generated features, models trained for 200 epochs with a batch size of 256, and input of 100,000 pairs.

The PCA baseline used a number of components sufficient to capture 95% of the variance in the training set for each data source (i.e. MALDI-TOF spectra and chemical fingerprints). The resulting projections are concatenated before being used to train logistic regression models. The results presented in the paper are obtained using the MACCS keys as chemical fingerprints.

The ResMLP configuration and training are kept constant in all experiments. The input projections encode the 6000-dimensional MALDI-TOF spectra and the 1024-dimensional Morgan fingerprints into 512-dimensional vectors, which are then concatenated. The model consists of 5 residual blocks, including ReLU activation, a linear layer of dimensionality 1024, a dropout layer with probability 0.2, and a BatchNorm layer. The ResMLP is trained with early stopping with a patience parameter of 50 epochs using an Adam optimiser with a learning rate of $3*10^{-4}$ and a weight decay of 10^{-5}. For the predictions on the single drug and species combinations, to compare to previous work, the model was additionally tuned with a reduced learning rate of 10^{-4} on the subset of samples for the target drug-species combinations that are not part of the test split for an additional 20 epochs.

Drug recommendation - Evaluation

To evaluate the recommendations produced, we use as metric the precision P, defined as the number of correct drugs recommended divided by the number of drugs in the intersection between the test data and the recommendation set. If no drug exists in the intersection, precision is set to 0. To analyse the effect of choosing different sizes for the recommendation set, we use the precision at cutoff n ($P@n$), which corresponds to the precision calculated for the top n recommendations. Finally, we computed the mean Average precision at cutoff n ($mAP@n$), an informative measure that considers not only the number of correct predictions but also the order of the recommended drugs. $mAP@n$ is the mean of the Average Precision at cutoff n, $AP@n$, calculated over all available queries. $AP@n$ is calculated as $AP@n = \frac{1}{\min(n, TP(total))} \sum_{i=1}^{n} \frac{TP(i) \cdot \mathrm{rel}(i)}{i}$, where TP stands for True Positives, and $\mathrm{rel}(i)$ is a binary indicator with value 1 if the ith item is relevant, 0 otherwise.

SHAP feature importance

To analyse the feature importance of the ResMLP model and verify the impact of the chemical fingerprints, we employed SHAP analysis Lundberg and Lee (2017). We used the DeepExplainer implementation of the Python `SHAP` package.

To interpret the importance assigned by the ResMLP to the chemical features, the model was trained with the same configuration as for the quantitative evaluation of the classification performance over 10 randomized train/test splits for each DRIAMS dataset. The overall importance of a feature is computed by averaging the absolute value of the SHAP value for that feature over the samples in the test set. The importance of a group of features is computed by first summing the SHAP values corresponding to the features for each sample and then calculating the overall importance of this sum as before.
